# Hypoxia-induced production of the cyclolipodepsipeptide BE-43547 by Micromonospora sp. RV43

**DOI:** 10.1099/mic.0.001662

**Published:** 2026-02-18

**Authors:** Marie Selch Tvilum, Camilla Bak Nielsen, Iben Steensgaard, Thomas Bjørnskov Poulsen, Mogens Johannsen, Thomas Tørring

**Affiliations:** 1 Department of Biological and Chemical Engineering, Aarhus University, Aarhus, Denmark; 2 Department of Forensic Medicine, Aarhus University, Aarhus, Denmark; 3 Department of Chemistry, Aarhus University, Aarhus, Denmark

**Keywords:** antibiotics, biosynthesis, hypoxia, secondary metabolites

## Abstract

The urgent need for new antibiotics emphasizes the importance of investigating the many biosynthetic gene clusters that remain silent under standard culture conditions and of assigning functions to the many natural products already discovered. Conventional approaches, such as altering the screening medium composition or culture conditions to trigger production, are often labourious and disconnected from the function of the natural products. In this study, we combine antimicrobial assays and the controlled culture conditions available in benchtop bioreactors to demonstrate that BE-43547, a natural product that exists as a mixture of congeners, is selectively active against Gram-positive bacteria growing anaerobically. Furthermore, we show that the producing organism *Micromonospora* sp. RV43 activates the biosynthesis of BE-43547 when cultured under low-oxygen conditions. These findings illustrate that linking compound function and regulation cues can provide a powerful strategy for discovering antibiotics with a new mode of action.

## Introduction

Natural products have been a major contributor to the success of human medicine, particularly in infectious diseases and cancer [1]. Moreover, recent studies have shown that considerable potential still exists in microbial sources [2]. A major challenge is the discrepancy between the number of biosynthetic gene clusters found in microbial genomes and the number of natural products detected when the same micro-organisms are cultivated in liquid media. They are also sometimes referred to as known unknowns [3] and pose several questions: are the biosynthetic gene clusters ever activated, and if so, what triggers this activation?

Many natural products are transcriptionally regulated through well-studied mechanisms and often tightly coupled to the lifecycle of the producing organisms [4]. A key example is the regulator DasR found in *Streptomyces* species. This is tightly coupled to a programmed cell death mechanism and elicits the production of secondary metabolites in response to sensing cell wall-derived *N*-acetylglucosamine [5,6]. These transcription factors and their cognate binding sites can also be used to predict new biosynthetic gene clusters and the chemical cues that might elicit the associated natural products [7].

In special circumstances, both the function and the chemical cues triggering the biosynthesis of the natural products are well studied, providing us with an understanding of why micro-organisms produce them. This is the case for siderophores – natural products produced under iron starvation, exported by the micro-organisms, and with specific receptor-mediated uptake once bound to iron [8]. This accumulated knowledge on biosynthesis and regulation has been exploited by researchers to identify new metallophores [9]. Studying similar relationships between function and regulation could guide the discovery of new molecules, triggers for activating cryptic biosynthetic gene clusters, and understanding the complex regulatory networks of natural product-producing micro-organisms.

**Fig. 1.**
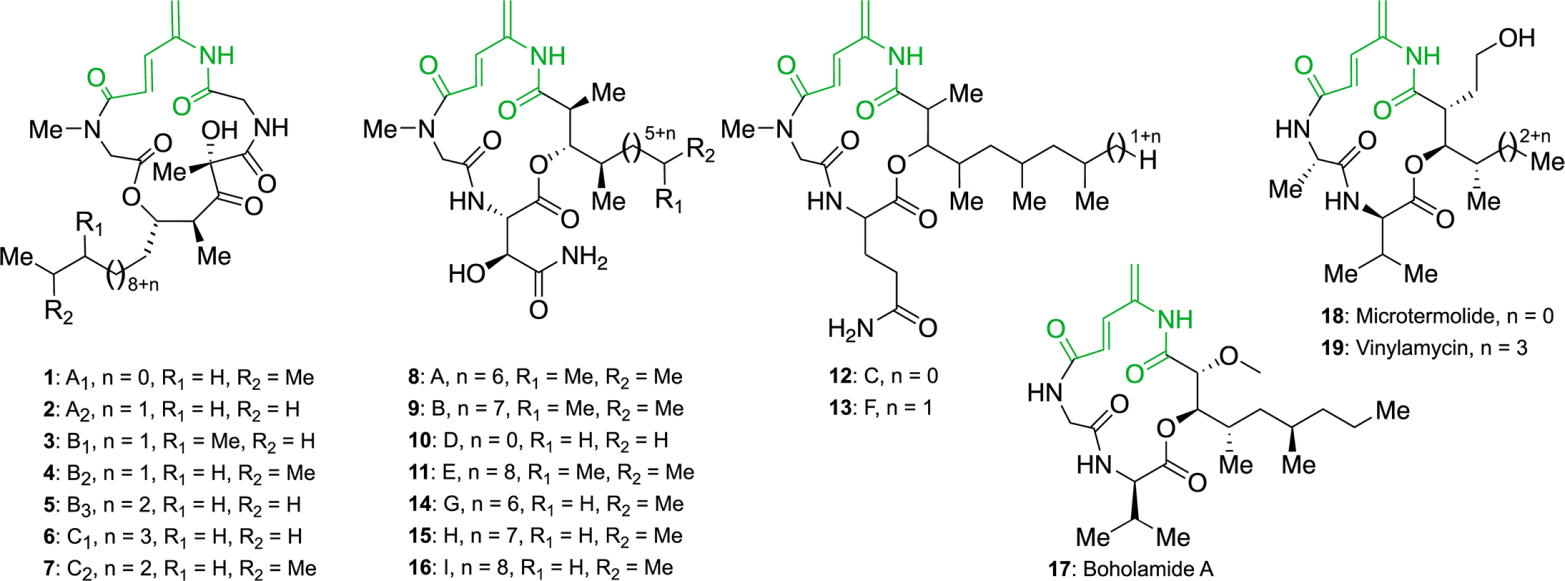
APD-CLD natural products. The APD moiety is shown in green. The structures shown are BE-43547 (1–7), rakicidins (8–16), boholamide A (17), microtermolide (18) and vinylamycin (19).

The natural products BE-43547 and rakicidins are part of the 4-amidopentadienoate cyclolipodepsipeptide (APD-CLD) family, along with microtermolide [10], vinylamycin [11] and boholamide [12] (Fig. 1). In general, they have been described to have weak or modest, if any, antibacterial effects except for the very potent activity against Gram-positive anaerobes reported for rakicidin G-I [13]. Several reports have described their hypoxia-selective cytotoxicity against cancer cell lines with selectivity indices of 4 (boholamide), 10 (rakicidin A/B) and 40 (BE-43547) (Fig. S1) [12,14,19]. This intriguing feature has created significant interest in the compounds and a demand for a reliable supply for biological testing. In our previous efforts to acquire enough BE-43547 for *in vivo* mice studies, we have encountered significant challenges with uneven and irreproducible production titres of BE-43547 from *Micromonospora* sp. RV43.

We hypothesized that the antibacterial activity would mirror the hypoxia-selective toxicity observed in cancer cell lines and that available oxygen might be involved in controlling the synthesis of the APD-CLD compounds in micro-organisms with APD-CLD-related BGCs. Herein, we show that the BE-43547 congeners and rakicidin A inhibit *Staphylococcus aureus* in a hypoxia-selective fashion and that fed-batch bioreactors with control over oxygen supply can be used to trigger the production of specialized metabolites from Actinomycetes.

## Methods

All micro-organisms and media recipes used in this study are listed in Tables S2 and S3.

### Antimicrobial activity testing

The congeners of BE-43547 and rakicidin A were isolated as previously described from *Micromonospora* sp. RV43 and *Micromonospora* sp. M42, respectively [15], but details can also be found in the Supplementary information (Figs S3–S5). Rakicidin D was isolated as described for rakicidin A, but from *Streptomyces lilacinus* NRRL B-1968. All compounds were used as purified.

### Minimal inhibitory and bactericidal concentrations

The antimicrobial activity of BE-43547, rakicidin A and rakicidin D was first explored by minimal inhibitory concentration (MIC) tests. A stock solution (DMSO, 1.6 mg ml^−1^) was used to make a 2-fold dilution series of each with Mueller–Hinton broth in 96-well plates. To here, one of the test strains (5×10^6^ c.f.u. ml^−1^) was added, all belonging to the ESKAPE pathogens, except for *Klebsiella oxytoca* substituting *Klebsiella pneumoniae* (ESKAPE: *Enterobacter cloacae* DSM 30054, *Pseudomonas aeruginosa* DSM 19880, *K. oxytoca* DSM 5175, *Acinetobacter baumannii* DSM 30007, *Enterococcus faecium* DSM 20477 and *S. aureus* DSM 20231). The plates were incubated either aerobically or anaerobically overnight (37 °C). If grown anaerobically, the plate was transferred to a sealed box containing a Microbiology Anaerocult A bag and incubated. The well with the lowest compound concentration without visual growth was set to be the MIC. All wells without visual growth were used to determine the minimal bactericidal concentration (MBC). In brief, the latter was determined by washing each well with media before a 2-fold dilution series was prepared in 0.9% NaCl water. From each dilution, 10 µl was spotted onto LB (lysogeny broth) agar plates and incubated overnight (37 °C). The well from which a spot contained five or fewer colonies was set as the MBC of said compound.

### Biofilm formation assay

To broaden the range of the BE-43547 antimicrobial activity, a biofilm assay [20] was conducted in 96-well plates. Here, a stock solution of BE-43547 (DMSO, 512 µg ml^−1^) was used to make a 2-fold dilution series in growth-promoting minimal M9 media with glucose and casamino acids (a final concentration at 1% of each). A peg-lid was inoculated with *S. aureus* DSM 20231 at 1×10^7^ c.f.u. ml^−1^ and combined with the above-mentioned 96-well plate before being incubated (72 h, 37 °C). The minimal biofilm inhibitory concentration (MBIC) and the minimal biofilm eradicating concentration (MBEC) were determined similarly to the MIC and MBC values, respectively.

### Persister cell killing assay

Adding to the antimicrobial activity of the BE-43547 compounds, we investigated their activity against dormant cells, using a persister cell (PC) killing assay in a 96-well plate as described elsewhere [21,22]. The same concentrations were used, as when determining the MIC, and tested against *S. aureus* DSM 20231 in minimal M9 media without carbon sources (i.e. glucose and casamino acids, final OD_600_ at 0.1) before being incubated anaerobically overnight (37 °C). Each well was used to construct a 10-fold dilution series, where all dilutions were spotted onto LB agar plates and incubated overnight (37 °C). The number of colonies was used to determine the c.f.u. ml^−1^ of *S. aureus*.

### Oxygen-dependent production of the APD-CLDs

#### Micro-organisms and media used

*Micromonospora* sp. RV43 [23] was used as the producing organism of the BE-43547 congeners (A_1_-A_2_, B_1_-B_3_ and C_1_-C_2_). For rakicidin production, *Micromonospora* sp. M42, *Micromonospora purpueogenes* NRRL B-2672, *Micromonospora chalcea* DSM 43026 and *Micromonospora eburnea* DSM 44814 were used. The cultivation media differed depending on the producing strain [ISP-2+22 g l^−1^ marine salts (*Micromonospora* sp. RV43, *M. purpueogenes* NRRL B-2672 and *Micromonospora* sp. M42) or A1M1 media (*M. chalcea* DSM 43026 and *M. eburnea* DSM 44814)]. If cultivated in bioreactors, a fed-batch protocol was used, where the feed was composed of either glucose (14 g l^−1^), yeast extract (4 g l^−1^) and marine salts (22 g l^−1^) (*M. purpueogenes*) or ISP-2, soy peptone (2 g l^−1^), starch (10 g l^−1^) and marine salts (22 g l^−1^) (*Micromonospora* sp. RV43 and *Micromonospora* sp. M42).

#### Inoculum preparations

The inoculum was prepared by adding mycelia stocks to their respective media in a ratio of 1:5 and incubated (2–3 days, 28 °C, 120–180 r.p.m, OD_600_=1.7–2.3). The inoculum was used to inoculate either bioreactors or shaker flasks in a ratio of 1:20.

Bioreactors (Infors Minifors II) were prepared by adding 2 l of their respective medium with a few drops of antifoam 204 before closing off all valves and openings. This was then sterilized. The temperature was adjusted to 28 °C, and the medium was saturated with oxygen before calibration of the oxygen sensor. The bioreactors were inoculated in the above-mentioned ratio.

#### Bioreactor cultivation

The bioreactors were first run as a batch production with a dissolved oxygen set-point at 20% pO_2_ for 48–72 h before being switched to a fed-batch production. During the fed-batch phase, two things were altered. The feed media were added (2 l) in a rate of 10% of maximum pump capacity (~20 ml h^−1^) unless stated otherwise, and if needed, the dissolved oxygen was adjusted to 5% pO_2_. Several parameters were fixed throughout production (Table 1). Four to five days after fed-batch initiation, the feeding ended. Productions ended after 8–10 days in total. To determine the dried cell weight (DCW) within each bioreactor, a 10 ml sample was centrifuged (4,000 r.p.m., 10 min), and the supernatant was decanted before washing the pellet (ratio 1:1 from original volume in water). The cell pellet was dried (60 °C, 2 days) and measured.

**Table 1.** Parameters and their settings of the bioreactors during the production of BE-43547 or the rakicidins

Parameter	Setting
pH	7.2 (±0.15)
Temperature	28 °C
Total flow	1–4 l min^−1^*
Stirrer speed	250–300 r.p.m.*

*If controlling the dissolved oxygen levels using nitrogen and pressurized air in the total flow, the total flow was kept constant at either 2 or 3 l min−1. Spanning in stirrer speed was still allowed, but at 300–350 r.p.m.

#### Feeding experiment

To investigate how the growth rate affects the production of BE-43547, we prepared four bioreactors according to the previously described method. The settings outlined in Table 1 used nitrogen to maintain dissolved oxygen levels at a constant 20% pO2 once the fed-batch phase began. We applied different feeding rates (5%, 7.5%, 10% and 15% of the maximum pump capacity) to simulate various growth rates, with the standard setup being 10%. Samples were taken out every 24 h, and the BE-43547 concentration was analysed as described below.

#### Shaker flask cultivation

Flasks with baffles were filled 2/5 of their volume with media. Like the bioreactors, shaker flasks were inoculated in a ratio of 1:20 and incubated in high-oxygen conditions (24–48 h, 180 r.p.m. and 28 °C). To simulate low-oxygen conditions, half of the shaker flasks were switched to 95 r.p.m. for the remaining time, having a total cultivation time of 7–8 days. Production of BE-43547 was evaluated every 24 h, and only the endpoint was analysed for the rakicidins. If necessary, 1,1-azobis(N,N-dimethylformamide) was added (1 or 2 mM) to the flasks at 180 r.p.m. after 24 h.

#### Extraction of the BE-43547 and rakicidins from flasks and bioreactors

For extraction, one of the following approaches was used: (1) the sample and acetonitrile were mixed in a ratio of 1:2, vortexed (30 s) and centrifuged (13,400 r.p.m., 5 min) before the supernatant was analysed using HPLC-MS (Agilent 1260 Infinity II). (2) The sample and ethyl acetate were mixed in a ratio of 1:1.2 and centrifuged (4,000 r.p.m., 10 min, 4 °C), and subsequently, the organic phase was dried in vacuo (SpeedVac, 40 °C). The sample was resuspended in MeOH in a ratio of 1:25 of the evaporated volume before being analysed using an HPLC-MS (Agilent 1260 Infinity II SQ). The capillary voltage was 135 V (ESI+). The nebulizing gas pressure was 15 psi, and the drying flow and temperature were 11 l min^−1^ and 300 °C, respectively. A reverse-phase column (Phenomenex Kinetex^®^ 5 µm C8 100 Å) with a total flow at 0.7 ml min^−1^ and a 5 µl injection volume was used to separate BE-43547 or the rakicidins. Two different gradients were used for separating either the congeners of BE-43547 or rakicidin A and B (Table 2). To determine the concentration of the product, either the area from the UV signal detected (260 nm) or the MS signal intensity was used. In this study, the total area of the BE-43547 or the rakicidins was used for estimating the concentration.

**Table 2.** Gradient used during HPLC-MS analysis during the extraction of BE-43547 or rakicidin A and B

BE-43547			Rakicidin A and B		
Time (min)	H_2_O+0.1% formic acid (%)	Acetonitrile+0.1% formic acid (%)	Time (min)	H_2_O+0.1% formic acid (%)	Acetonitrile+0.1% formic acid (%)
0	30	70	0	95	5
1	30	70	2	95	5
10	5	95	4	40	60
12	5	95	15	5	95
13	30	70	18	5	95
15	30	70	19	95	5
			22	95	5

#### Metabolomics of *Micromonospora* sp. RV43 incubated in flasks

Using a similar approach as described above, we cultivated the *Micromonospora* sp. RV43 in a shaker flask setup, using triplicates of each condition. From this, 5 ml samples were taken after 24, 48 and 120 h, and the pellets were separated before being frozen. Flasks were shifted to 95 r.p.m. after 24 h if needed.

After thawing the pellets, they were worked up by the addition of 80% ice-cold methanol, i.e. 1.4 ml for the 24 h samples, 2.3 ml for the 48 h samples and 6.0 ml for the 120 h samples, and mixed for 5 min. From the mixed samples, a 200 µl aliquot was pipetted into an Eppendorf tube and left for 10 min at 4 °C prior to centrifugation (10 min, 10,000 r.p.m.). Supernatants were transferred into Eppendorf tubes, and the pellet was extracted an additional time with 200 µl of 80% ice-cold methanol. The combined extracts were vacuum-centrifuged (SpeedVac, 40 °C) until dry and resuspended in 600 µl Milli-Q water with 0.1% formic acid prior to LC-MS analysis. A QC sample was prepared by pooling equal volumes of all samples.

Untargeted metabolomics and downstream univariate and multivariate data analysis were done as previously described [24]. In brief, the LC-MS system consisted of UPLC-QTOF with an ACQUITY I-Class UPLC (Waters Corporation, Milford, MA, USA) equipped with an ACQUITY UPLC HSS T3 column (2.1 mm×100 mm, 1.8 µm, Waters) and coupled to a Bruker maXis Impact QTOF mass spectrometer (Bruker Daltonics, Bremen, Germany). The samples were analysed in random order with an injection volume of 10 µl in ESI− and 2 µl in ESI+. QC samples were injected at the beginning, followed by every fifth sample. The mobile phase consisted of Milli-Q water with 0.1% formic acid (A) and a mixture of MeOH/ACN (1:1 v/v) with 0.1% formic acid (B). The flow rate was set to 0.4 ml min^−1^ at a column temperature of 50 °C with the following 21 min gradient: 0% B (0–2 min), 0–40% B (2–6 min), 40–60% B (6–6.5 min), 60–88% B (6.5–11 min), 88–100% (11–11.5 min), 100% B (11.5–18 min), 100–0% B (18–19.5 min) and 0% B (19.5–21 min). Features were extracted using XCMS (version 4.4.0) [25]. The centWave algorithm [26] was used for peak picking with a resolution of 12 ppm and a signal-to-noise threshold of 6. The Obiwarp algorithm was used for retention time correction [27]. Gap filling was conducted to recover missing signals in the raw data. Features had to be present in at least 80% of the samples within one specific sample group to be considered for further analysis. Isotopes, adducts and ion source fragments were annotated using CAMERA (version: 1.62.0) [28]. Coefficients of variation (CV) of QC samples were calculated to omit features with CV >30%. After analysis, each sample was normalized to the total peak intensity in each group (time points). The areas from the XCMS results were exported to SIMCA (version 18.0.0.372, Sartorius Stedim Data Analytics AB, Göttingen, Germany) for multivariate data analysis. Principal component analysis (PCA) was used as quality control to show the potential cluster trend of the groups and QC samples. Subsequently, orthogonal projections to latent structures discriminant analysis (OPLS-DA) was used to assess the global change between groups, high and low oxygen. Pareto scaling was applied for both PCA and OPLS-DA. Variable importance in projection (VIP) scores were calculated from OPLS-DA, providing a score for how different the two groups at low and high oxygen were at three different time points (24, 48 and 120 h). Fold changes were calculated as mean intensity ratios between groups, high and low oxygen, at the same time points. Variance equality was tested with an F-test, followed by two-sample t-tests (assuming equal or unequal variances as appropriate). Features were regarded as statistically significant when the *P*-value<0.05, the fold change >20% and the VIP score >1. It should, however, be noted that the number of replicates was limited in the setup, suggesting that some findings could be false positives. Finally, for identification, m/z value (<5 ppm), retention time and fragments were compared with an in-house database containing authentic standards. Metabolites identified at level 2 were compared to the MetFrag database by m/z value (<5 ppm) and fragments.

## Results and discussion

### BE-43547 and rakicidin A are hypoxia-selective antibiotics against *S. aureus*

**Fig. 2.**
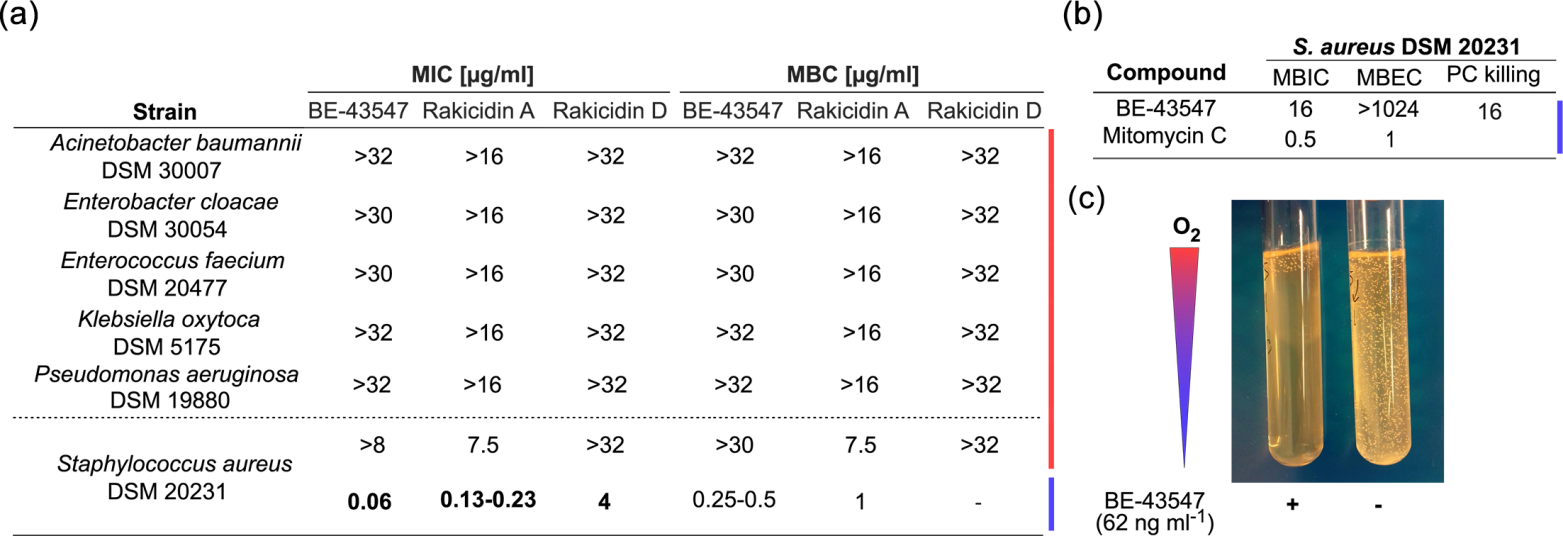
The bioactivity of BE-43547, rakicidin A and rakicidin D against the ESKAPE pathogens. (**a**) MIC and MBC values tested either aerobically (red) or anaerobically (blue). If no bioactivity was observed within the tested concentrations, it is indicated with a ‘>’ symbol. (**b**) Biofilm assays and PC killing against *S. aureus*. Mitomycin C was the control compound against biofilm formation*.* (**c**) Visualization of the hypoxia-selective activity of BE-43547 (62 ng ml^−1^, left) and its growth control (right) when tested against *S. aureus* DSM 20231 in thioglycolate broth. An oxygen gradient is created throughout the tube as indicated by the bar (red=atmospheric, blue=anaerobic)*.*

The BE-43547 congeners have no previously reported antibacterial activity, and we decided to test BE-43547, rakicidin A and rakicidin D on the ESKAPE pathogens (with *K. oxytoca* instead of *K. pneumoniae*) using standard conditions for MIC determination. The tested BE-43547 sample consists of a mix of all seven congeners, which differ in length and branching of the acyl chain, and comparable activity against cancer cell lines [29]. The results (Fig. 2a, red) show that rakicidin D has no antibacterial effect in the tested concentrations up to 32 µg ml^−1^, while rakicidin A showed modest activity against *S. aureus* with an MIC value of 7.5 µg ml^−1^ and a minimum bactericidal concentration (MBC) of 7.5 µg ml^−1^. When testing BE-43547, we noticed a decrease in growth compared to the controls starting at low µg ml^−1^ quantities. However, it never extended below visible growth (<0.07 absorbance units), which was further supported by the tested MBC values as they showed viable cells even at the highest concentration (Fig. S2). The same trend was observed for *E. faecium*, but not for any of the Gram-negative pathogens. Since *S*. *aureus* is a facultative anaerobe, we also repeated the MIC and MBC determinations under anaerobic conditions. This revealed a dramatic change in MIC of 0.06, 0.2 and 4 µg ml^−1^ for BE-43547, rakicidin A and rakicidin D, respectively (Fig. 2a, blue and Fig. S2), mirroring the hypoxia-dependent bioactivity previously described for cancer cells [14,17]. This change also affected the MBC values for BE-43547 (0.5 µg ml^−1^) and rakicidin A (1 µg ml^−1^). This is a very rare phenotype that is not observed for common antibiotics like vancomycin but is known from metronidazole, a drug used to treat infections caused by *Clostridium difficile* and other anaerobic organisms [30]. It also compares well to a previous report by Chen *et al*., who demonstrated potent activity of rakicidin G-I against Gram-positive anaerobes, such as *C. difficile* and *Peptostreptococcus anaerobius* [13].

We also investigated the effect of the compounds on PCs of *S. aureus* using a starvation-based assay and found a modest effect (Fig. 2b). The effect on *S. aureus* biofilm as reflected in the minimum biofilm inhibitory concentration (MBIC) and minimum biofilm eradication concentration (MBEC) values was also modest (Fig. 2b). To illustrate the stark difference in effect on aerobic and anaerobically growing *S. aureus*, we inoculated *S. aureus* into thioglycolate broth with and without 0.06 µg ml^−1^ of BE-43547. The thioglycolate acts as a reducing agent to create an oxygen gradient throughout the tube, leaving high oxygen concentrations at the air-liquid interface and anoxia near the bottom of the tube. The results are shown in Fig. 2(c), which highlights the hypoxia-dependent growth inhibition, making *S. aureus* appear as an obligate aerobic bacterium.

### *Micromonospora* sp. RV43 produces BE-43547 in increased titres when cultivated at low oxygen concentrations

**Fig. 3.**
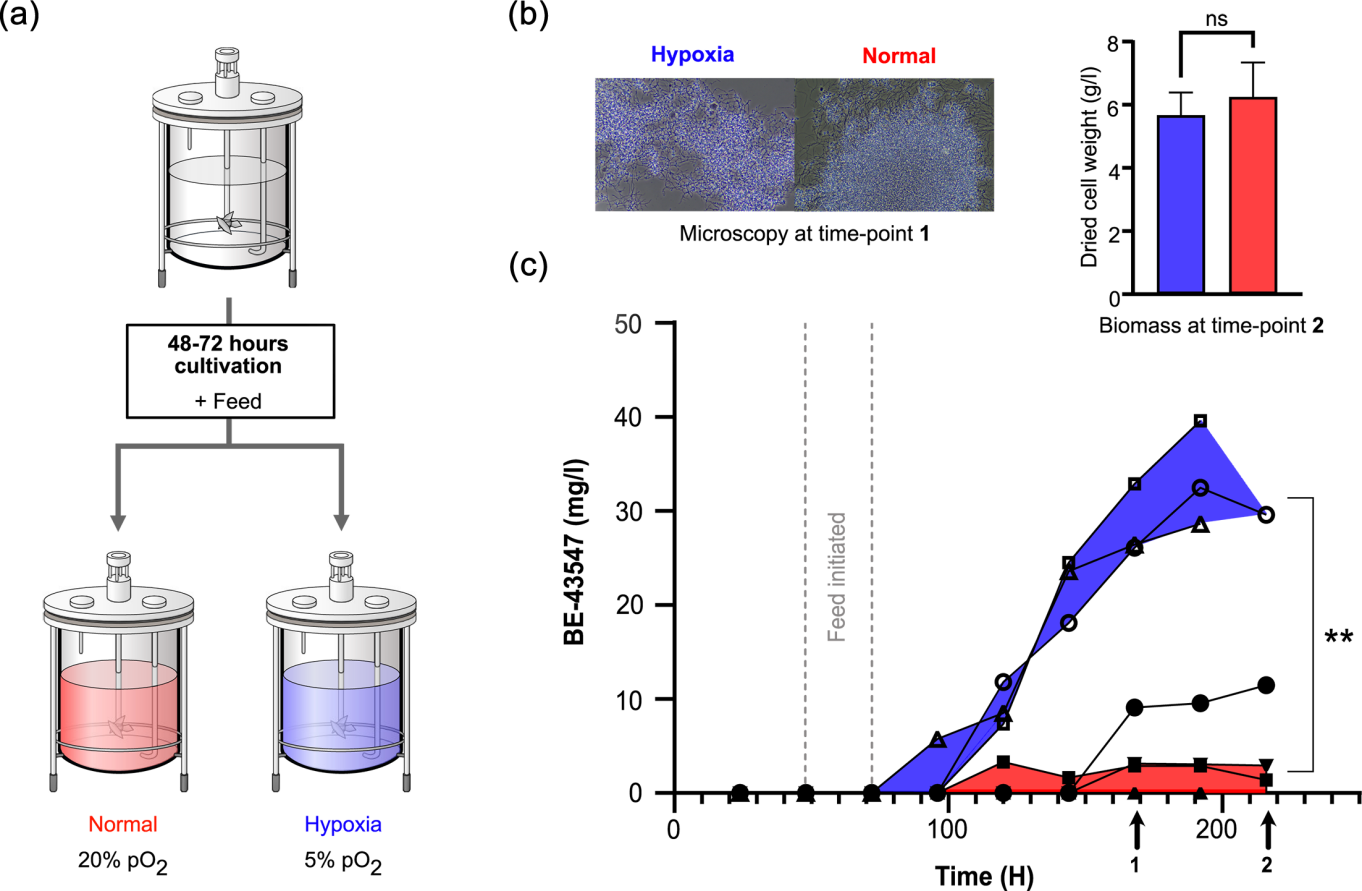
Production of BE-43547 congeners by *Micromonospora* sp. RV43 using benchtop bioreactors. (**a**) All bioreactors with *Micromonospora* sp. RV43 were kept at a setting of 20% pO_2_ (red) for the first 48–72 h after inoculation. After this initial batch period, feeding was initiated (pump speed: 10%) for all bioreactors, before splitting them into two subgroups: half were kept at 20% pO_2_ (red) while half were changed to 5% pO_2_ (blue) for the remainder of the cultivation. (**b**) The DCW (g/l) from bioreactors with 5% pO_2_ (blue) and 20% pO_2_ (red). The data are presented as the mean of biological triplicates±sd. A two-tailed unpaired t-test showed a non-significant difference between the two settings regarding DCW (ns, *P*>0.05). Representative microscopy pictures of *Micromonospora* sp. RV43 at the two oxygen levels after 168 h. (**c**) Combined production of the BE-43547 congeners (mg/l) at 5% pO_2_ (blue) and 20% pO_2_ (red) during production. Open shapes indicate a setting of 5% pO_2_, and filled shapes indicate a setting of 20% pO_2_. The total area from all congeners detected at 260 nm on an HPLC-MS was used to find the total concentration shown. The shaded area of blue and red highlights the range in which we find the BE-43547 production at 5% or 20% pO_2_, respectively, from which the black filled circles are excluded from the latter due to a failure in keeping the pO_2_ levels at a constant 20%. Each graph represents a biological replicate, and the concentration is determined by extractions done in technical duplicates, which are presented here as the mean. The concentration of each extraction is shown in Table S4. ** indicates a significant difference (*P*<0.005) between the two conditions, when using the BE-43547 concentrations after 192 h, determined by a two-tailed unpaired t-test*.*

Inspired by the hypoxia-dependent antibiotic effect of the BE-43547 and rakicidins, along with previous struggles with uneven and unpredictable production titres, we decided to investigate whether oxygen availability during cultivation had any effect on the production titres. We speculated that if the observed hypoxia-dependent bioactivity had any ecological relevance, it could be repressed by the surplus of oxygen that we typically strive for in laboratory settings, whereas hypoxic stress might serve as a cue for production initiation. Also, because the oxygen supply can be difficult to control or even monitor in shaker flasks, it could result in the unreliable production levels we had experienced for this strain. As *Micromonospora* and *Streptomyces* species are generally considered obligate aerobes, we had to strike a balance in oxygen supply that is difficult to achieve or control in shaker flasks. Previous work by Gallagher *et al*. on the *Streptomyces* strain CNQ-525 in a chemostat used air saturation values of 20 and 5%, respectively, to simulate high and low oxygen environments and trigger a shift in the metabolic output of the napyradiomycin biosynthetic gene clusters [31]. We, therefore, shifted to a set of benchtop bioreactors to cultivate one of the producing organisms, *Micromonospora* sp. RV43, in fed-batch mode. A fed-batch approach has the advantage of providing a prolonged exponential or deceleration phase, while both monitoring and controlling the pH and dissolved oxygen concentration. The bioreactors contained an initial volume of 2 l (ISP-2 medium with marine salt). After inoculating, two feedback loops were started: one maintaining the pH at 7.2 (with a deadband of 0.15) by allowing the addition of acid (1 M H_2_SO_4_) and base (1M NaOH), and the other maintaining a dissolved oxygen concentration (pO_2_) of 20% by allowing a change in airflow (1–4 l min^−1^) and, if insufficient, stirrer speed (250–300 r.p.m.). After the batch phase (48–72 h), we initiated feeding (ISP-2 medium with marine salt, peptone and starch) and either maintained the available oxygen at 20% throughout the feeding phase or lowered it to 5% to simulate hypoxic stress (see setup in Fig. 3a). We designed the stirrer speed window (250–300 r.p.m.) to be as narrow as possible to minimize any effect of increased shear stress. By sampling daily, we could monitor the cellular morphology using microscopy and determine the concentration of BE-43547 by HPLC-MS (Figs S3, S6 and S8). We observed no notable difference in the morphology (Figs 3b and S8a), but, as seen in Fig. 3(c), a drastic increase in production follows the hypoxic stress induced by lowering the available oxygen concentration. By controlling the oxygen concentration, we can achieve more than 40 mg l^−1^ by the end of the fermentation. This clearly demonstrates a correlation between low oxygen availability and the production of the BE-43547 compounds. A recent study by Devine *et al*. found that the production of formicamycin was repressed by the redox-sensitive regulator ForJ in aerated liquid cultures, indicating that this might be a more broadly applied strategy [32]. We also measured DCW from all bioreactors after finishing the fermentation but observed no significant difference, indicating that the bacteria are still accumulating biomass to a similar extent despite the lower amount of dissolved oxygen concentration (Fig. 3b). Additionally, we observed an induced production, though at lowered levels, if the bioreactors went below our 20% pO_2_ setting point for a shorter period (filled black circles in Fig. 3c). To ensure the production was not affected by an increased shear stress, we repeated the experiment using a nitrogen cascade to control the oxygen levels within the bioreactors, thereby keeping our inlet flow and stirrer speed constant. This resulted in the same tendency of induced production during low oxygen levels compared to normal oxygen levels (Fig. S7).

**Fig. 4.**
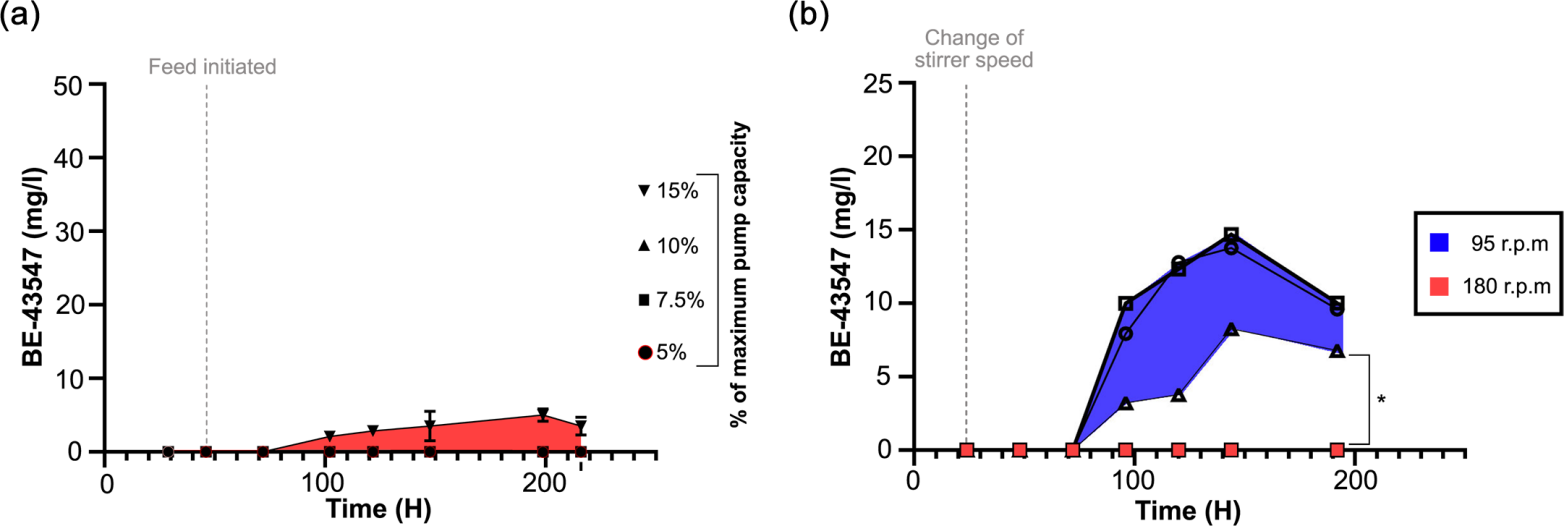
Inferred growth rate and shaker flask production of BE-43547. (**a**) Production of BE-43547 dependence on feeding rate. A series of bioreactors was inoculated and allowed to accumulate biomass at 20% pO_2_. After 48 h, feeding was initiated at a variable pump speed. The data are presented as the means of triplicate extractions, as indicated by error bars, and the red shaded area highlights the range in which we find the BE-43547 production at 20% pO_2_. (**b**) Flask production of BE-43547 from *Micromonospora* sp. RV43, having 180 r.p.m. for 24 h, followed by a switch to 95 r.p.m. (blue) or kept constant at 180 r.p.m. (red). Each graph represents a biological replicate, and the concentration is determined by extractions done in technical duplicates, which are presented here as the mean. The concentration of each extraction is shown in Table S5. The blue shaded area highlights the range in which we find BE-43547 production at 5% pO_2_. * indicates a significant difference (*P*<0.05) between the two conditions, when using the BE-43547 concentrations after 200 h, determined by a two-tailed unpaired t-test*.*

Secondary metabolites are generally assumed to be produced in the deceleration or stationary phase, when the specific growth rate of the bacteria decreases from the µ_max_ of the exponential phase. To investigate if the production of BE-43547 is more generally coupled to a decrease in specific growth rate and therefore only indirectly to the oxygen concentration, we screened several different feeding rates, as this controls the specific growth rate in a fed-batch reactor. All the experiments were conducted with the high pO_2_ setting, and none showed the sharp production increase observed for low pO_2_ settings (Fig. 4a). Again, this indicates that BE-43457 is controlled by oxygen limitation and not a lowered specific growth rate. We could also replicate the differential production in shaker flasks by using the shaking speed as a crude way of creating low and high oxygen conditions in a simple batch experiment, and without monitoring the dissolved pO_2_. As previously observed, this gave a larger variation, but still a clear difference between the two conditions (Fig. 4b).

### The hypoxia-triggered production is unique for the BE-43547 producer

**Fig. 5.**
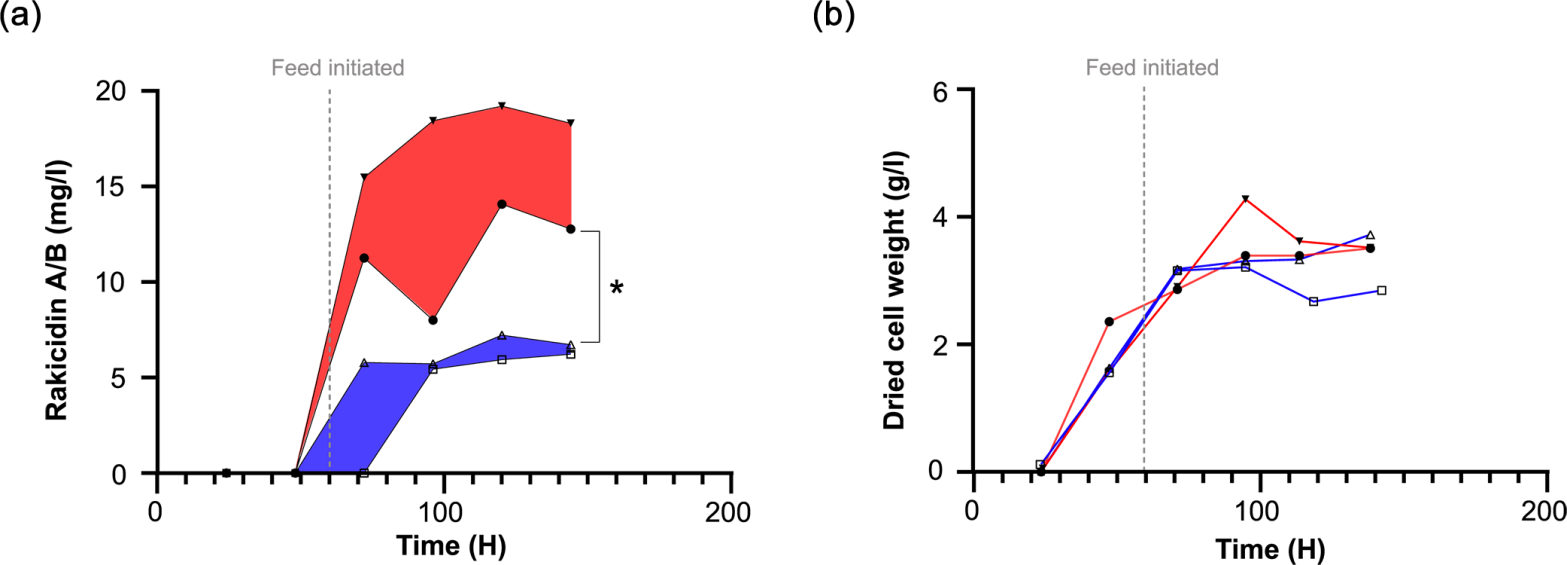
Rakicidin A and B production by *Micromonospora* sp. M42 using bioreactors. (**a**) Combined production of rakicidin A and B using *Micromonospora* sp. M42 with a setting of 20% pO_2_ for 60 h, before switching half to 5% pO_2_ (blue) while the remaining were kept constant at 20% pO_2_ (red). Subsequently, additional nutrients are added at a 10% rate of maximum pump capacity. Open shapes indicate a setting of 5% pO_2_, and filled shapes indicate a setting of 20% pO_2_. The total area from rakicidin A and B detected at 260 nm on an HPLC-MS was used to find the total concentration shown. The shaded area of blue and red highlights the range in which we find the rakicidin production at 5% or 20% pO_2_, respectively. Each graph represents a biological replicate, and the concentration is determined by extractions done in technical duplicates, which are presented here as the mean. The concentration of each extraction is shown in Table S6. * indicates a significant difference (*P*<0.05) between the two conditions, when using rakicidin A and B concentrations after 144 h, determined by a two-tailed unpaired t-test. (**b**) Biomass development during production. Each graph represents a biological replicate, and the biomass is determined from technical duplicates and presented here as the mean value.

The rakicidins are another group of APD-CLD compounds produced by various *Micromonospora* strains, and like BE-43547, some rakicidins are hypoxia-selective cytotoxins and also have potent activity against anaerobic bacteria [13]. While the difference in bioactivity between normoxic and hypoxic conditions is not as pronounced as for BE-43547, we were still intrigued to investigate if the production of rakicidins was also induced by hypoxic stress. To test this, we used a set of four bioreactors and repeated the previous experiment, but this time using *Micromonospora* sp. M42, a strain we previously established as a producer of rakicidin A and B [14]. Analysing samples drawn daily using HPLC-UV-MS to determine the amount produced, it is clear from Fig. 5(a) that the opposite trend is observed. As previously mentioned, the growth based on accumulated DCW is not significantly affected by the low oxygen.

**Fig. 6.**
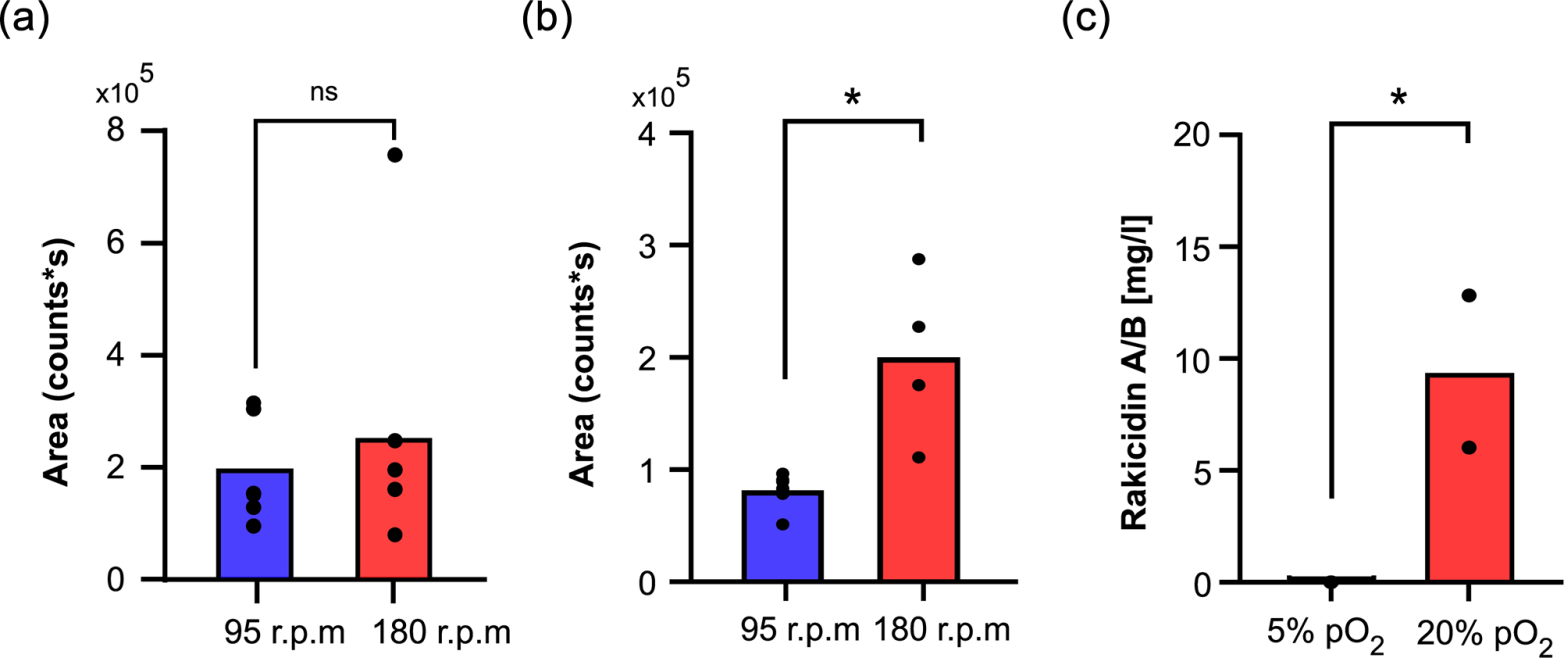
Production of rakicidin A/B on the last day of production using either Erlenmeyer flasks (**a, b**) or bioreactors (**c**). Similar setup as for the flask experiment from *Micromonospora* sp. RV43 (Fig. 4b) with the switch moved from 24 to 48 h, or as the bioreactor setup from *Micromonospora* sp. M42 (Fig. 5). Area (count*s) is based on EIC of 607.4 m/z and 621.4 m/z. The concentrations of rakicidin A/B in (c) are based on the UV signal measured at 260 nm. (**a**) *M. chalcea* DSM 43026. (**b**) *M. eburnea* DSM 44814. For (a) and (b), the data are presented as the means of biological replicates (*n*=4–6) at 95 r.p.m. (blue) and 180 r.p.m. (red). (**c**) *M. purpueogenes* NRRL B-2672. The data are presented as the means of biological duplicates. Each is extracted in technical duplicates, and the mean value of these is represented as the data points, at either 5% or 20% pO_2_. * indicates a significant difference (*P*<0.05) between the two conditions at the end of production, determined by a two-tailed unpaired t-test; ns indicates a non-significant difference.

To ensure that this was not an isolated case, we also tested three additional strains: the first strain, *M. purpueogenes* NRRL B-2672, which we had previously shown to produce rakicidin A and B, and two additional strains, *M. chalcea* DSM 43026 and *M. eburnea* DSM 44814, which we had bioinformatically predicted to be rakicidin producers [33], in a simplified flask setup (Fig. 6a, b) and benchtop bioreactors (Fig. 6c). We confirmed their ability to produce both rakicidin A and B using HPLC-MS, but we observed the same trend of higher shaking speed leading to increased production titres as seen in *Micromonospora* sp. M42 (Figs S9 and S10).

### Low oxygen affects relatively few metabolites in *Micromonospora* sp. RV43

To get insights into the cellular responses when exposing the *Micromonospora* sp. RV43 to an oxygen-limited environment, we did a metabolic profiling of the intracellular metabolites. We used the same setup as shown in Fig. 4(b), and after 120 h of incubation, we identified metabolites that were more (blue) or less (red) abundant in the flasks at low stirrer speed compared to the ones at high stirrer speed (Figs S7 and S11). As expected, this clearly shows an increased abundance of the BE-43547 compounds. It should be noted that the statistics are based on triplicates, which could have been strengthened with more replicates.

**Fig. 7.**
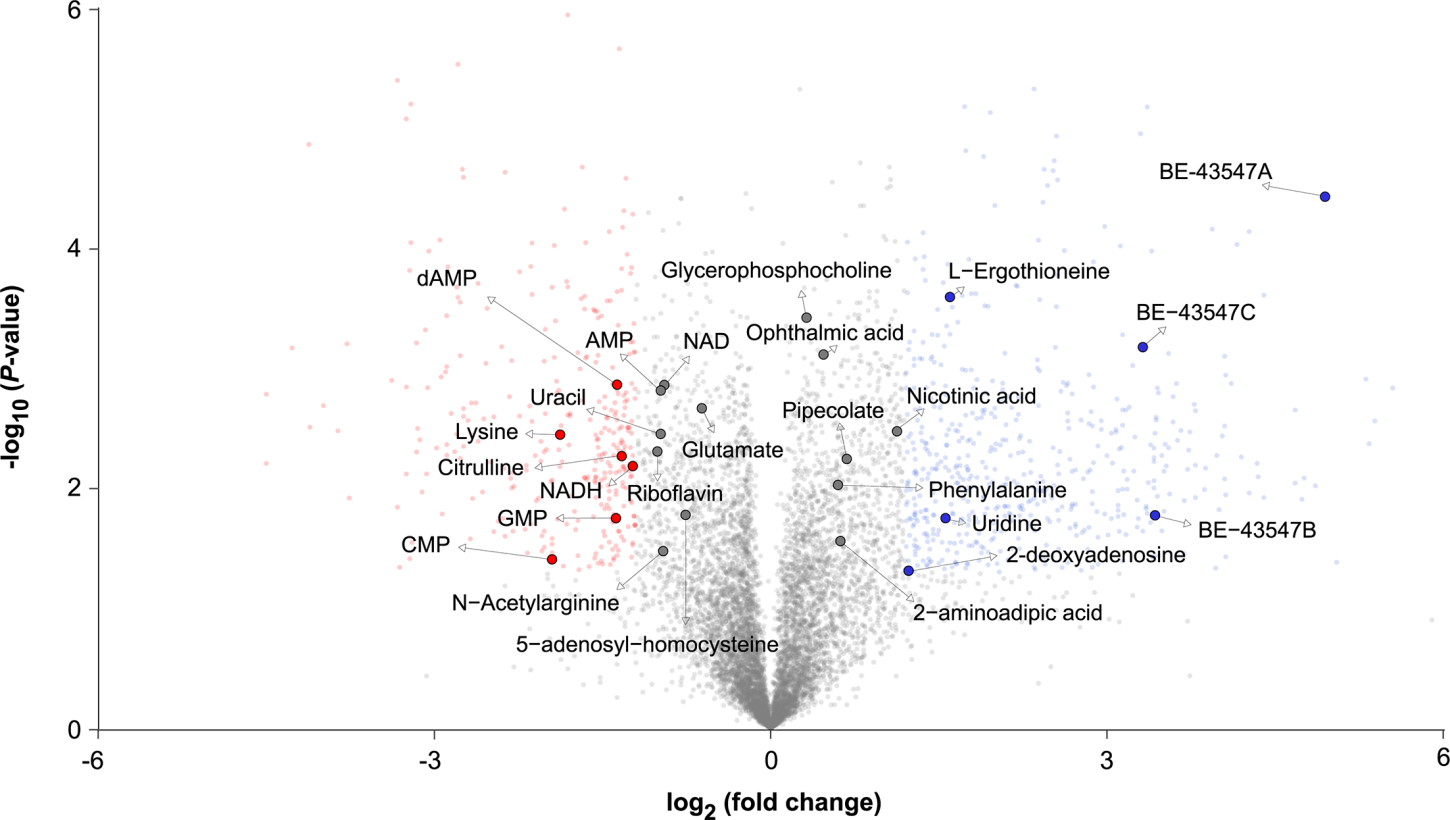
Responses of metabolites putatively identified from oxygen stress after 120 h of cultivation using positive electrospray ionization mode. Using the same setup as shown in Fig. 4(b), the metabolites from low oxygen levels are compared to normal oxygen levels. The metabolites during low oxygen cultivations are found in increased (blue) or decreased (red) abundance based on certain parameters (log2 fold change=1.2, *P*-value<0.05). Metabolites identified and with statistically increased or decreased abundance based on XCMS are highlighted (fold change <0.8 or >1.2, *P*-value<0.05 and VIP score >1)*.*

The amino acid levels were not affected to the same extent as previously observed in actinobacteria grown under saline-induced [34] stress or iron starvation [35]. Here, phenylalanine and 2-aminoadipic acid were found to accumulate during lowered oxygen levels. Both glutamate and citrulline, which are affiliated with the arginine pathway, were found in low intensities, indicating a shift towards arginine biosynthesis during low oxygen availability. On the contrary, we found no increased abundance of arginine after 120 h, although increasingly abundant after 48 h of incubation (Table S1). We do not consider samples from 24 h, as these have been subjected to the same oxygen levels and are therefore assumed to be similar. This is further supported by the PCA plots (Fig. S11). When looking at the nucleotide derivatives, we find the monophosphates AMP, GMP and CMP at low intensities. Additionally, adenosine, cytidine, guanosine and uridine are all increased at 48 h, whereas only uridine remains increased after 120 h. In a previous study, the authors found a similar profile of nucleotide derivatives increasing during salinity-induced stress of *Streptomyces coelicolor*, which could suggest that this type of stress-induced regulation is more general to Actinomycetes and not specific to *Micromonospora* sp. RV43 and its BE-43547 production. Combining the changes in amino acid-related metabolites, and nucleotides and derivatives, we find no direct connection to low oxygen levels or BE-43547 production. Similarly, when we look at the major thiol buffer in Actinomycetes, mycothiol, there is no alteration in its abundance (Table S1). Mycothiol, in combination with ergothioneine, is known for its ability to help sustain a highly reducing environment. Here, the latter has previously been shown to play a role in protecting *S. coelicolor* from oxidative stress [36]. We do observe a pronounced effect on ergothioneine with increased abundance under hypoxic conditions, which could suggest it plays a similar role in our producing organism.

The metabolic profile shows that the low oxygen conditions appear to affect the production of the BE-43547 more pronouncedly than the primary metabolism and less so than similar studies on salinity- or iron-induced stress.

### Chemically induced oxidative stress does not increase titres

Intrigued by the production feature unique to the *Micromonospora* sp. RV43 and the relatively modest impact of low oxygen on the primary metabolism, we set out to investigate how oxygen depletion could selectively induce BE-43547 production. Using antiSMASH, we searched through the BGC to identify regulatory elements known to be affected by oxygen but found nothing that would explain our phenomenon (Fig. S12) [33]. Oxidative stress is a term covering multiple stress responses, including disulphide and redox stress, which can be present at high and low levels of oxygen. While actinobacteria are generally regarded as obligate aerobes, some, like *Mycobacterium tuberculosis*, also cope with long periods of low oxygen levels [36]. A key cellular component is sensor proteins, which typically contain reactive thiols or metal centres that can respond to oxidative stress. One described example is RsrA, which normally binds the σ^R^ factor in *S. coelicolor* [37]. The σ^R^ factor is a global transcriptional regulator that controls >100 genes, including several involved in anti-oxidant roles. Normally, RsrA binds and inactivates σ^R^, but upon oxidative stress, a complex set of reactions in RsrA results in the release of σ^R^ [37]. This, in turn, triggers a cellular response to oxidative or disulphide stress [38,39]. We speculated whether the transcription of the BE-43547 encoding BGC was directly or indirectly affected by oxygen level or whether oxidative stress in general would elicit the biosynthesis through regulation like that of σ^R^. We used a similar flask setup to the one previously described, where the *Micromonospora* sp. RV43 was incubated at high oxygen conditions. We then chemically triggered oxidative stress, in the form of disulphide stress, after 24 h by adding the oxidizing agent 1,1-azobis(N,N-dimethylformamide), but found no induced production of the compounds (Fig. S13). An explanation could be that σ^R^ activates the expression of a pathway-specific repressor at high oxygen levels and during oxidative stress. Another possibility could be a redox-sensitive repressor, similar to the ForJ regulator in the formicamycin BGC [32,40]. ForJ forms a disulphide-mediated tetramer under high oxygen concentrations which represses the transcription of the formicamycin biosynthetic genes. Under low oxygen concentration, the disulphides are reduced, and ForJ dissociates into dimers, enabling transcription and formicamycin biosynthesis. Upon inspection of the vicinity of the BE-43547 BGC, we found no similar regulators (Fig. S12), but we do observe genes that are seemingly pathway-specific. Future studies will determine if they are involved in the regulation.

In combination with the metabolic profile, this leaves the molecular mechanism behind the regulation an open question for future research. But the correlation of hypoxia-coupled bioactivity and hypoxia-induced production creates a strong incentive for screening micro-organisms under oxygen-limiting conditions in addition to the more widely used OSMAC [41] approaches, such as medium composition, and in combination with bioactivity tests under hypoxia.

## Conclusion

A major challenge in natural product discovery is the known unknowns. These are the BGCs we can bioinformatically predict but not couple to natural products produced under laboratory conditions. In this study, we demonstrate a hypoxia-selective bioactivity of different APD-CLD members against *S. aureus*, with potencies of 0.06, 0.2 and 4 µg ml^−1^ for BE-43547, rakicidin A and rakicidin D, respectively. Interestingly, this mirrors the hypoxia-selective cytotoxicity that has previously been reported in cancer cells. Supported by a bioreactor setup, we simulated low oxygen levels during cultivation of the BE-43547 producer *Micromonospora* sp. RV43 and found a significant increase in production compared to those producing at normal oxygen levels, with the highest concentrations reaching above 40 mg l^−1^. The increased production was not related to growth rate, changes in morphology or biomass concentrations. Lastly, we observed no direct connection between the hypoxia-selective bioactivity of rakicidin A and B and its production conditions. The increased production of BE-43547 in a hypoxia-dependent manner is, to the best of our knowledge, unique to the *Micromonospora* sp. RV43, leaving the understanding of how oxygen depletion affects the strain yet to be elucidated. To explore this matter, we chemically induced oxidative stress, activating the σ^R^ factor known to be activated during oxidative stress, but seemingly without affecting the production. Furthermore, we carried out metabolic profiling to gain insights into the cellular response during limited oxygen availability. Based on the primary metabolites we annotated, we find no major perturbation that can explain the connection between the induced production of BE-43547 and oxygen limitations. This combination suggests that the BE-43547 compounds are controlled by a specific regulatory mechanism, which needs further investigation to be elucidated. One limitation of this analysis is that many secondary metabolites are secreted, while we analysed the intracellular metabolites. Therefore, future studies could be expanded to whole cultures or supernatants to provide a more detailed insight into the secreted metabolites.

While we can only speculate on the ecological implications, we have noted that the *Micromonospora* sp. RV43 was isolated from a Mediterranean marine sponge, *Aplysina aerophoba* [23], that, when kept in aquaria, will stop its water pumping for extended periods of time. Previous research, describing the measured oxygen concentration in the surface and in the tissue of the sponge, has determined that anoxia sets in after ~15 min of non-pumping [42]. Combined with our results, this could provide the sponge with control over the chemical arsenal of its microbiome.

## Supplementary material

10.1099/mic.0.001662 Fig. S1.

## Abbreviations

APD-CLD: 4-amidopentadienoate cyclolipodepsipeptide
CV: coefficients of variation
MBC: minimal bactericidal concentration
MBEC: minimal biofilm eradicating concentration
MIC: minimal inhibitory concentration
OPLS-DA: orthogonal projections to latent structures discriminant analysis
PC: persister cell
PCA: principal component analysis
VIP: variable importance in projection
