# Supplementary material for: Hypoxia-induced production of the cyclolipodepsipeptide BE-43547 by Micromonospora sp. RV43
Source: Microbiology (Reading). 2026 Feb 18;172(2):001662. doi: 10.1099/mic.0.001662 (PMC12916061; doi:10.1099/mic.0.001662)
Supplement: Fig. S1. [file mic-172-01662-s001.pdf]

Supplementary Information

Hypoxia-induced Production of the Cyclolipodepsipeptide BE-43547 by *Micromonospora* sp. RV43

*Marie Selch Tvilum[1], Camilla Bak Nielsen[2], Iben Steensgaard[1], Thomas Bjørnskov Poulsen[3], Mogens Johannsen[2], Thomas Tørring[1]**

1. *Department of Biological and Chemical Engineering, Aarhus University, Aarhus, Denmark*
2. *Department of Forensic Medicine, Aarhus University, Aarhus, Denmark*
3. *Department of Chemistry, Aarhus University, Aarhus, Denmark*

**Corresponding author – thomast@bce.au.dk*

Structure

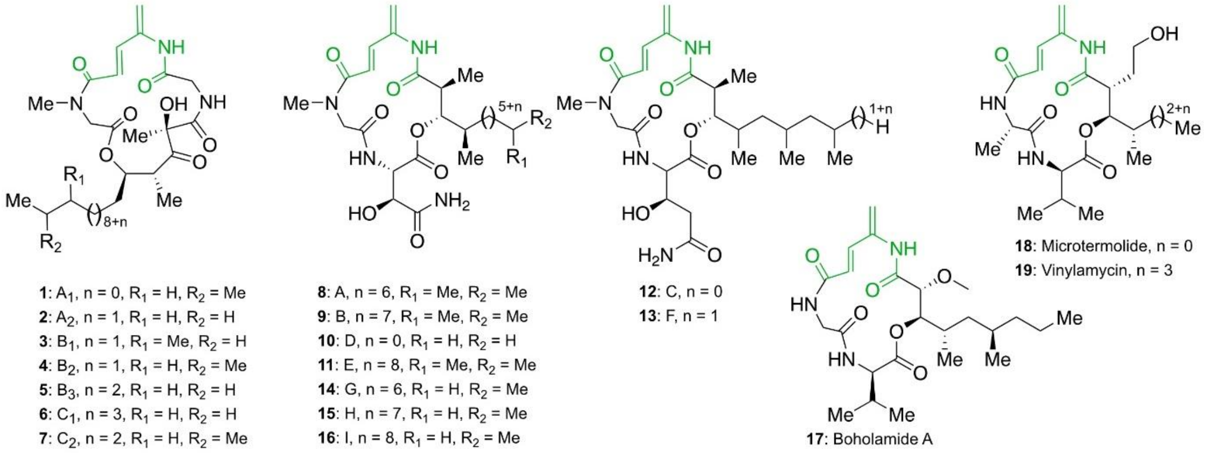

Bioactivity

1: PANC-1 cells (IC_{50} = 41 nM)[1]

2: PANC-1 cells (IC_{50} = 53 nM)[1]

1-7: *Staphylococcus aureus* DSM 20231 (MIC = 0.06 µg/mL, MBC = 0.25 µg/mL, MBIC = 16 µg/mL, Persister cell killing = 16 µg/mL)[This study]

8: PANC-1 cells (IC_{50} = 62 nM),[1] M109 cells (IC_{50} = 40 ng/mL),[2] Gram-positive bacteria** (MIC = 1.0 µg/mL, MIC = 4.2 µg/mL, MIC = 2.1 µg/mL, MIC = 2.1 µg/mL, MIC = 4.1 µg/mL, MIC = 4.1 µg/mL), L929 cells (MIC = 1.2 µg/mL),[3] *Staphylococcus aureus* DSM 20231 (MIC = 0.13-0.23 µg/mL, MBC = 1 µg/mL)[This study]

9: M109 cells (IC_{50} = 200 ng/mL),[2] HCT-8 cells (IC_{50} = 430 µM), PANC-1 cells (IC_{50} = 486 µM),[4] Caco-2 cells (IC_{50} = 457 µM),[4] Gram-positive bacteria** (MIC = 1.0 µg/mL, MIC = 4.2 µg/mL, MIC = 4.1 µg/mL, MIC = 2.1 µg/mL, MIC = 4.1 µg/mL, MIC = 4.1 µg/mL), L929 cells (MIC = 1.2 µg/mL)[3]

10: Carcinoma colon 26-L5 (IC_{50} = 3.3 µg/mL),[5] *Staphylococcus aureus* DSM 20231 (MIC = 4 µg/mL)[This study]

11: HCT-8 cells (IC_{50} = 19.3 ng/µL), PANC-1 cells (IC_{50} = 9.96 ng/µL), anaerobic bacteria* (MIC = 16 µg/mL, MIC = 32 µg/mL, MIC = 2 µg/mL, MIC = 2 µg/mL, MIC = 8 µg/mL), [6] Gram-positive

bacteria** (MIC = 1.0 µg/mL, MIC = 4.2 µg/mL, MIC = 8.3 µg/mL, MIC = 16.5 µg/mL, MIC = 8.3 µg/mL, MIC = 4.1 µg/mL), L929 cells (MIC = 1.2 µg/mL)[3]

12: *Bacillus subtilis* PCI219 (MIC = 50 µg/disc)[7]

13: *Bacillus subtilis* PCI219 (MIC = 50 µg/disc), *Escherichia coli* ATCC 25922 (MIC = 25 µg/disc)[7]

14: HCT-8 cells (IC_{50} = 20.7 ng/µL), PANC-1 cells (IC_{50} = 9.2 ng/µL), anaerobic bacteria* (MIC = 1 µg/mL, MIC = 1 µg/mL, MIC <= 0.125 µg/mL, MIC = 0.5 µg/mL, MIC = 1 µg/mL)[6]

15: HCT-8 cells (IC_{50} = 19.4 ng/µL), PANC-1 cells (IC_{50} = 8.7 ng/µL), anaerobic bacteria* (MIC = 8 µg/mL, MIC = 8 µg/mL, MIC = 1 µg/mL, MIC = 2 µg/mL, MIC = 4 µg/mL)[6]

16: HCT-8 cells (IC_{50} = 17 ng/µL), PANC-1 cells (IC_{50} = 7.8 ng/µL), anaerobic bacteria* (MIC = 4 µg/mL, MIC = 8 µg/mL, MIC = 1 µg/mL, MIC = 2 µg/mL, MIC = 1 µg/mL)[6]

17: U87MG glioblastoma cells (IC_{50} = 120 nM)[8]

****Gram-positive bacteria**[3]
Micrococcus luteus DSM 1790, *Bacillus subtillis* DSM 10, *Staphylococcus aureus* Newman, *Staphylococcus aureus* DSM 11822 MRSA, *Enterococcus faecium* DSM 20477, *Enterococcus faecium* DSM 17050 VREF

***Anaerobic bacteria**[7]
Clostridium difficile ATCC 43255, *Clostridium difficile* ATCC BAA-1382, *Peptostreptococcus anaerobius* ATCC 27337, *Porphyromonas gingivalis* ATCC BAA-308, *Propionibacterium acnes* ATCC 11827

Fig. S1: All APD-CLD compounds and their bioactivity. The values in blue indicate that it was retrieved during hypoxic/anaerobic conditions. The APD moiety is shown in green. The structures shown are BE-43547 (**1-7**), the rakicidins (**8-16**), Boholamide A (**17**), Microtermolide (**18**), and Vinylamycin (**19**) natural products.

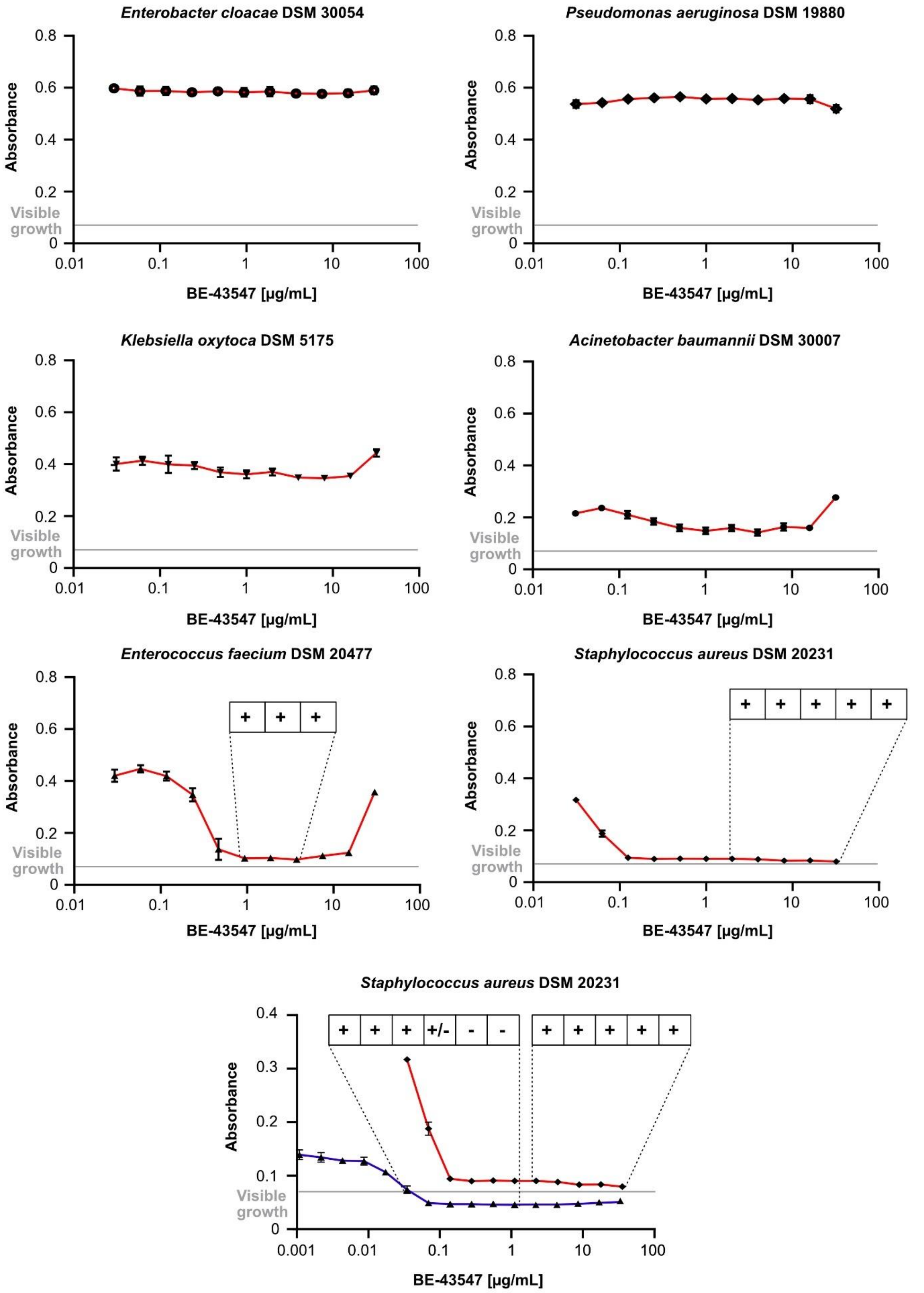

Fig. S2: BE-43547 MIC assays against the ESKAPE pathogens tested aerobically (red) or anaerobically (blue). MIC value is determined when no visible growth is found, which is indicated at an absorbance value of 0.07. Concentrations tested for

MBC are shown in boxes, resulting in either a biostatic effect (+) or a biocidal effect (-). The pathogens tested are indicated above each graph.

Production and purification of the BE-43547 congeners, rakicidin A and D

BE-43547: Seed cultures were prepared in Erlenmeyer flasks (500 mL, with baffles and steel strings) filled with ISP2 with Instant Ocean (200 mL) and inoculated with spores of *Micromonospora* sp. RV43. The cultures were incubated (250 rpm, 28°C) until an optical density of approximately 0.5.

Six Erlenmeyer flasks (5 L with baffles), each containing 1 L M8 with Instant Ocean, were inoculated (approx. 100 mL). The cultures were monitored for production daily and harvested after 5-8 days of incubation (130 rpm, 28°C). The cultures were either extracted with ethyl acetate or by incubation overnight with Dianion HP-20 resin. The crude product was subsequently fractionated over an open reverse-phase column (LiChroprep RP-C18). Fractions containing the compounds were reduced *in vacuo* and purified on a preparative Agilent 1260 Infinity II using an Eclipse XDB-C8 column (Agilent, 250 × 21.2 mm, 7 µm). The relevant fractions were combined and dried *in vacuo*.

Rakicidin A: Seed cultures were prepared in Erlenmeyer flasks (500 mL, with baffles and steel strings) filled with ISP2 with Instant Ocean (200 mL) and inoculated with spores of *Micromonospora* sp. M42. The cultures were incubated (250 rpm, 28°C) until an optical density of approximately 0.5.

Six Erlenmeyer flasks (5 L with baffles), each containing 1 L ISP2 with Instant Ocean, were inoculated (approx. 100 mL). The cultures were monitored for production daily and harvested after 5-8 days of incubation (130 rpm, 28°C). The cultures were either extracted with ethyl acetate or by incubation overnight with Dianion HP-20 resin. The crude product was subsequently fractionated over an open reverse-phase column (LiChroprep RP-C18). Fractions containing the compounds were reduced *in vacuo* and purified on a preparative Agilent 1260 Infinity II using an Eclipse XDB-C8 column (Agilent, 250 × 21.2 mm, 7 µm). The relevant fractions were combined and dried *in vacuo*.

Rakidicin D: was prepared using the same workflow as for rakicidin A, but with *Streptomyces lilacinus* NRRL B-1968.

a)

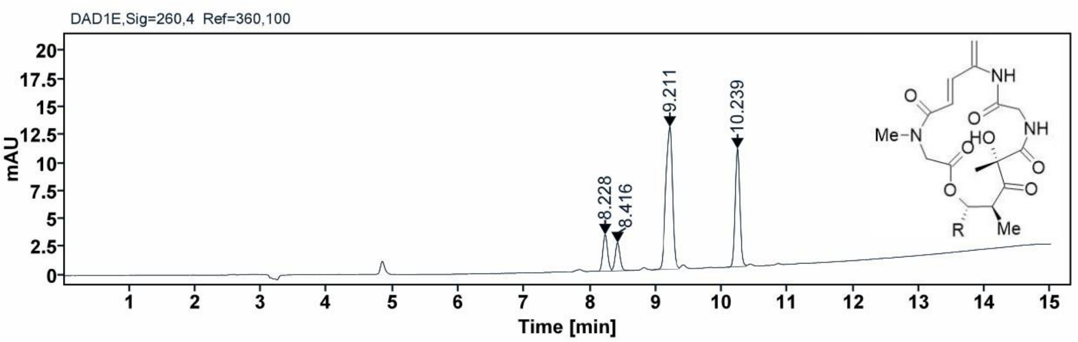

b)

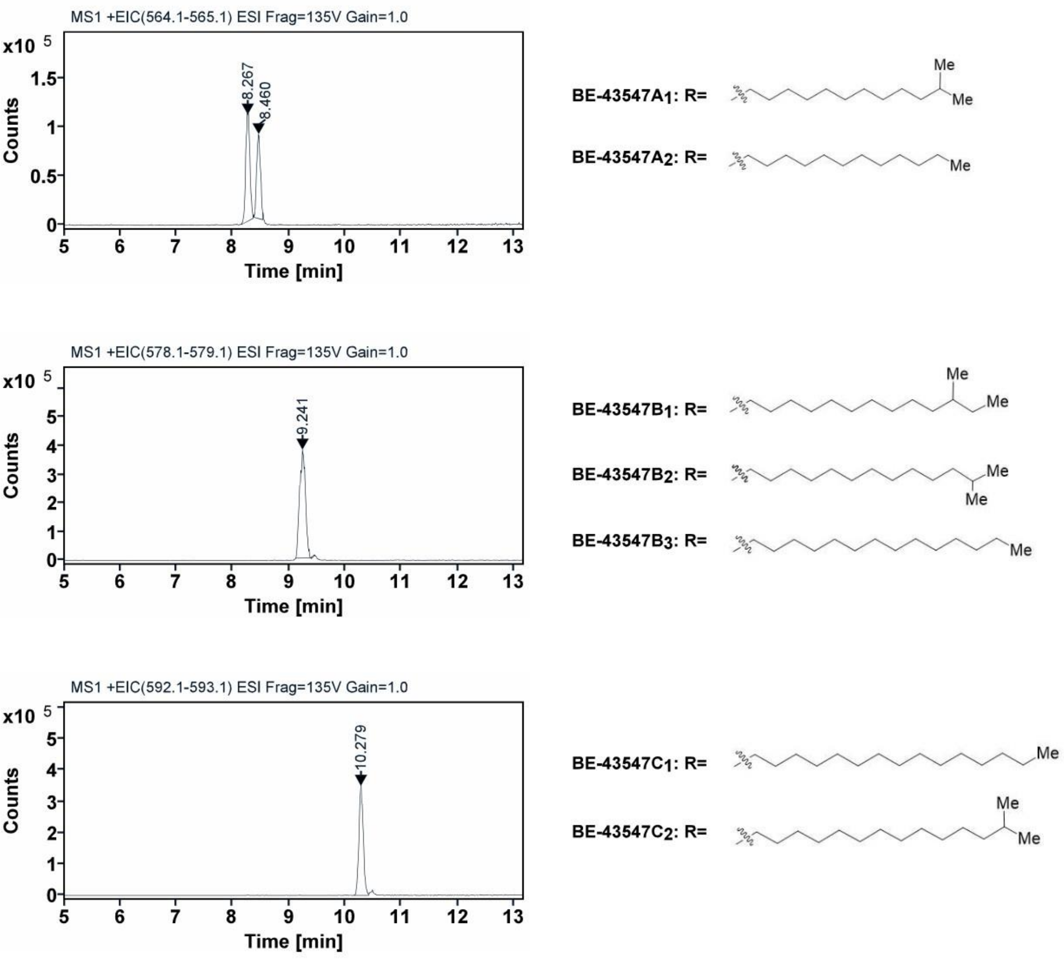

Fig. S3: Chromatogram at 260 nm of the BE-43547 congeners purified from production by *Micromonospora* sp. RV43. a) Ultraviolet signals from HPLC (260 nm) of 12.5 µg/mL BE-43547 stock with a 5 µL injection. The core structure of the BE-43547 is shown on the right, where the R-group indicates the type of congener. b) Extracted ion currents (EIC) traces at 260 nm with masses matching those of the BE-43547 congeners, showing that the peaks from a) consist of these congeners. The sidechains of each congener are shown on the right. The BE-43547A_1-A_2 (top) was separated during elution. It has not been possible to separate BE-43547B_1-B_3 (middle) and the BE-43547C_1-C_2 (bottom) congeners from each other, and it is therefore assumed that each peak is a mix of the congeners, which has been shown previously.[1]

a)

b)

c)

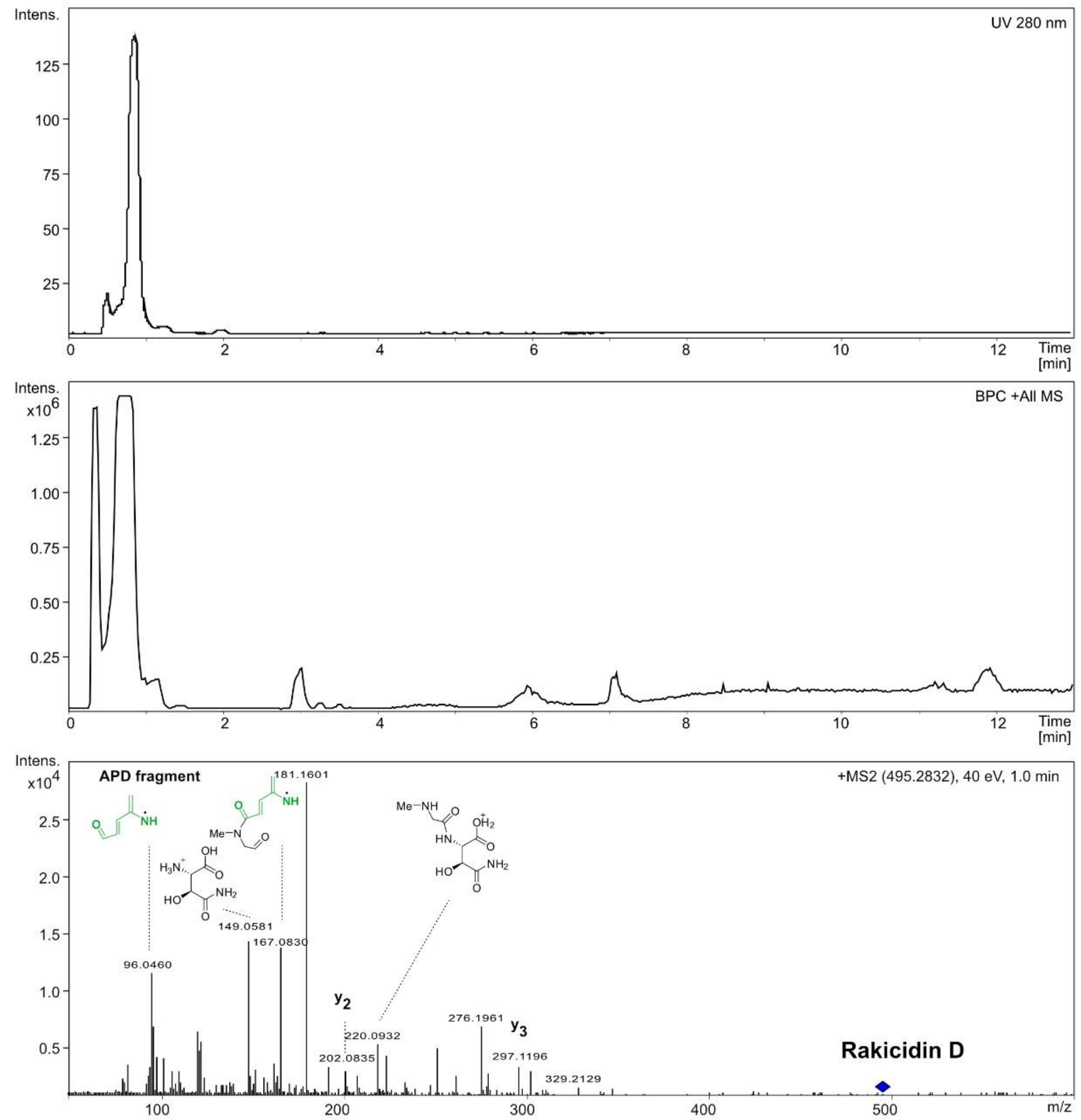

d)

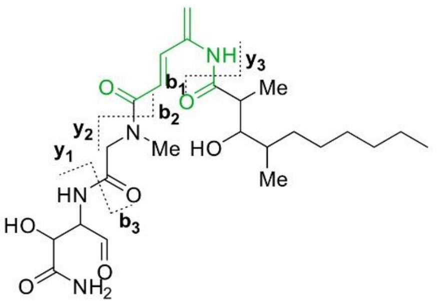

e)

Ion formula	[M+H]⁺ Theo.	[M+H]⁺ Obs.	Mass error (ppm)
$C_{24}H_{39}N_4O_7^+$	495.2813	495.2832	-3.8362
$C_{12}H_{17}N_4O_5^\bullet$ (**y_3**)	297.1199	297.1196	1.0097
$C_{17}H26NO_2^+$	276.1964	276.1961	1.0862
$C_7H_{14}N_3O_5^+$	220.0928	220.0932	-1.8174
$C_7H_{12}N_3O_4^\bullet$ (**y_2**)	202.0828	202.0835	-3.4639
$C_{12}H_{21}O^+$	181.1586	181.1601	-8.2800
$C_8H_{11}N_2O2^\bullet$	167.0821	167.083	-5.3866
$C_4H_9N_2O_4^+$	149.0557	149.0581	-16.1014
$C_5H_6NO^\cdot$	96.0449	96.0460	-11.4530

Fig. S4: HPLC-MS/MS of rakicidin D. a) UV chromatogram at 280 nm. b) base peak chromatogram from MS. c) MS/MS spectrum with some fragment ions shown. The capillary voltage was 40 eV (ESI+). d) Annotation of fragment ions after spontaneous ring-opening of the ester bond. e) Table of fragment ions, the theoretical and observed m/z value along with the mass error (ppm). The annotated fragments are consistent with previously reported ones.[1,2]

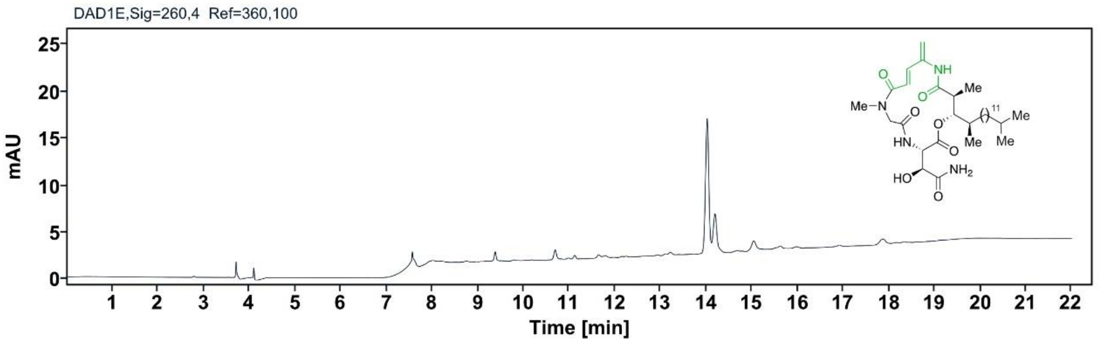

Fig. S5: HPLC of rakicidin A. UV chromatogram at 260 nm. Rakicdin A elutes after 14 minutes.

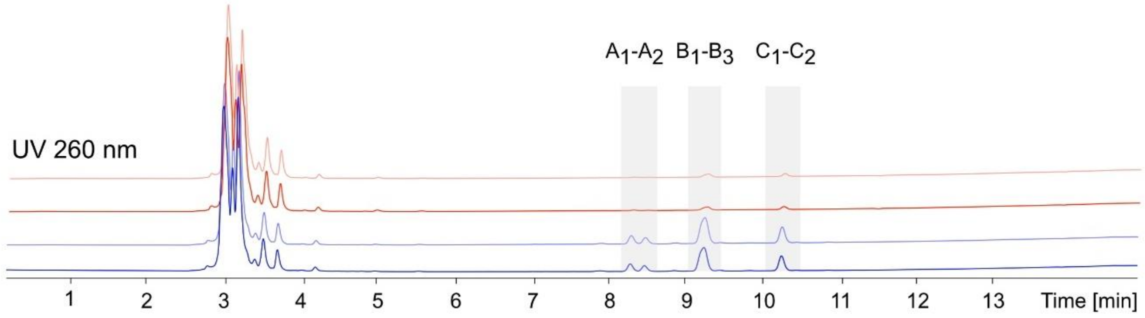

Fig. S6: **Representative UV chromatograms at 260 nm of the BE-43547 production from *Micromonospora* sp. RV43**. Samples were extracted after 8 days of production in bioreactors. Five µl from each sample were injected onto a C8 column in an HPLC-MS system. Each sample was extracted in technical duplicates from two bioreactors, where the chromatograms are shown for all extractions. Production was conducted either at limited (5% pO_2, blue) or high (20% pO_2, red) oxygen levels.

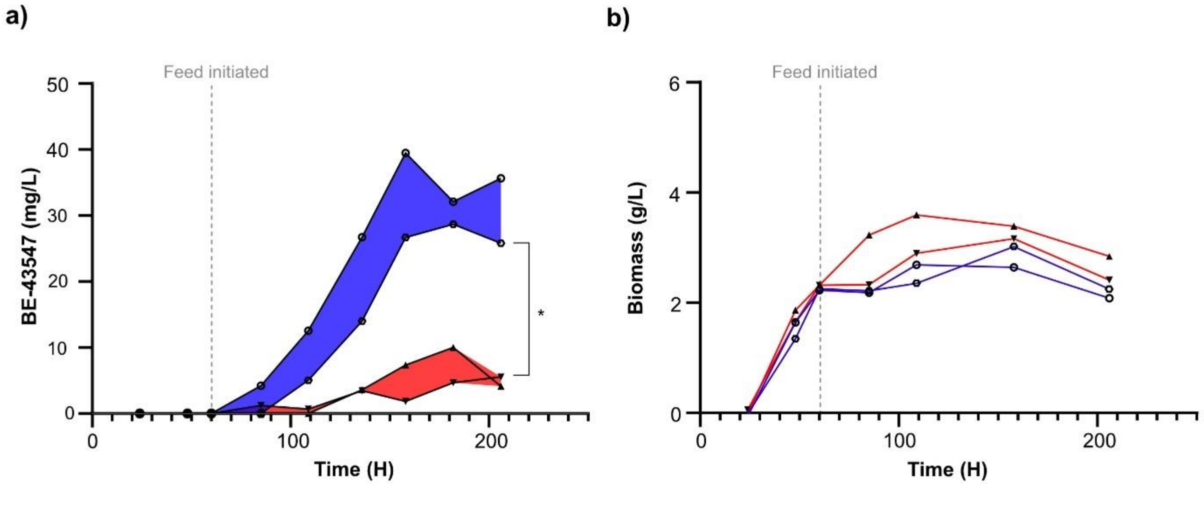

Fig. S7*:* Production of BE-43547 congeners by *Micromonospora* sp. RV43 using bench-top bioreactors with a nitrogen cascade to control oxygen levels. The oxygen levels were controlled by a combination of nitrogen and atmospheric air, and keeping the stirring speed between 250-300 rpm and a total flow of 3 L/min. a) Production of the BE-43547 congeners (mg/L) at 5% pO_2 (blue) and 20% pO_2 (red). Open shapes indicate a setting of 5% pO_2, and filled shapes indicate a setting of 20%

pO_2. The total area from all congeners detected at 260 nm on an HPLC-MS was used to find the total concentration shown. The shaded area of blue and red highlights the range in which we find the BE-43547 production at 5% or 20% pO_2, respectively. Each graph represents a biological replicate, and the concentration is determined by extractions done in technical duplicates, which are presented here as the mean. The concentration of each extraction is shown in **Table S7**. * indicates a significant difference ($P < 0.05$) between the two conditions, when using the BE-43547 concentrations after 210 hours, determined by a two-tailed unpaired t-test. b) The biomass development during cultivation. Each graph represents a biological replicate, which is extracted in technical duplicates and here presented as the mean.

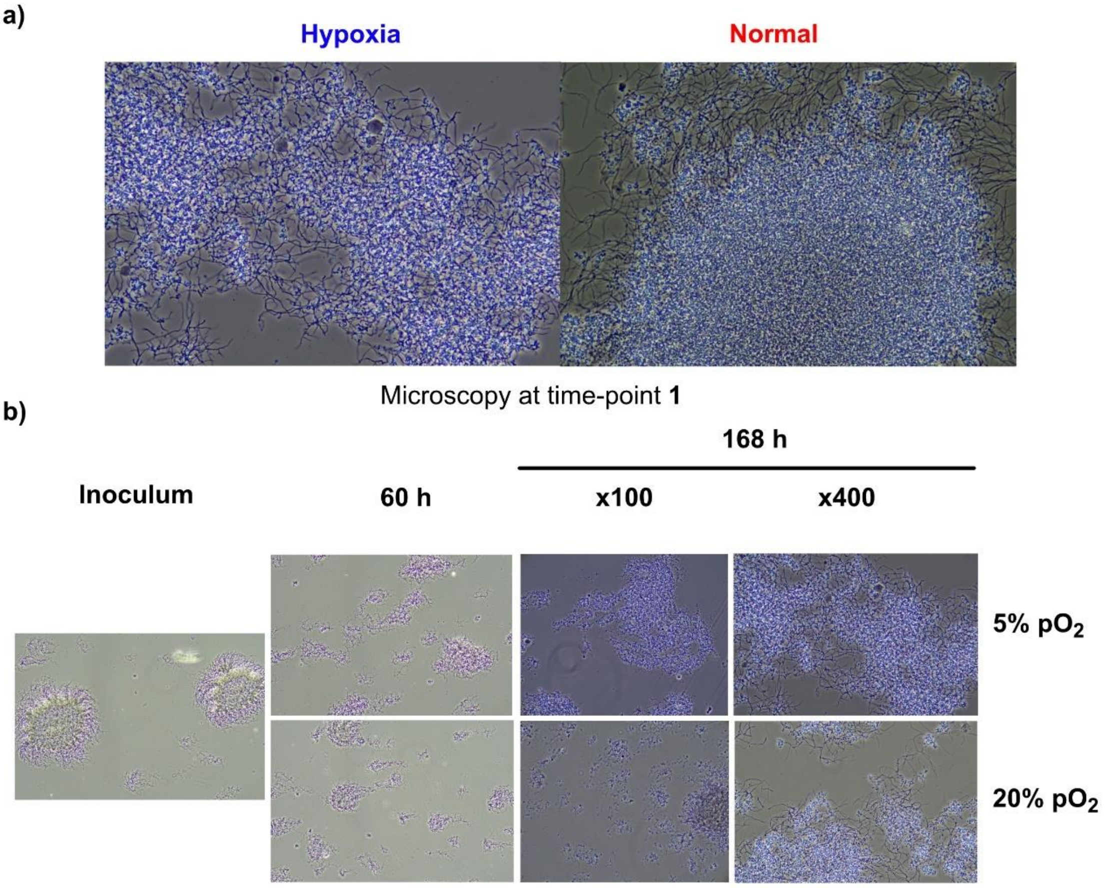

Fig. S8: Microscopy pictures of *Micrmonospora* sp. RV43 during production of BE-43547 in bioreactors at low (blue, 5% pO_2) or high (red, 20% pO_2) levels of dissolved oxygen. a) An enlarged figure of Fig. 3b. b) The time points taken for microscopy are the inoculum used, after 60 hours of production, and after 168 hours of production. All are shown with a factor 100 enhancement, except for the ones at the far right, which are at 400 times enhancement.

a)

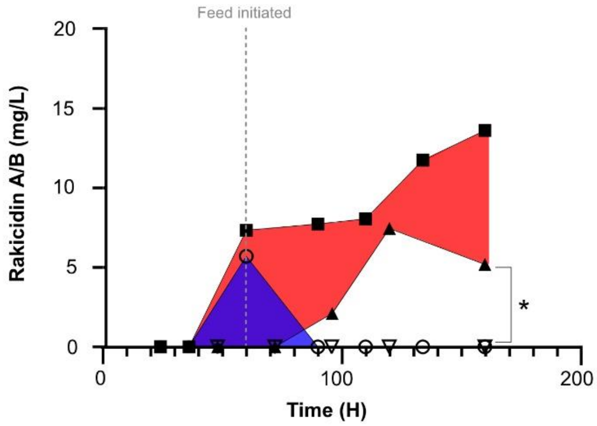

Fig. S9: Production of rakicidin A and B by *Micromonospora purpueogenes* NRRL B-2672 using bench-top bioreactors. The development of Rakicidin A and B titers during production, with nutrients added from 60 hours onwards. Open shapes indicate a setting of 5% pO_2, and filled shapes indicate a setting of 20% pO_2. The total area from rakicidin A and B detected at 260 nm on an HPLC-MS was used to find the total concentration shown. The shaded area of blue and red highlights the range in which we find rakicidin A and B production at 5% or 20% pO_2, respectively. Each graph represents a biological replicate, and the concentration is determined by extractions done in technical duplicates, which are presented here as the mean. The concentration of each extraction is shown in **Table S8**. * indicates a significant difference ($P < 0.05$) between the two conditions, when using the rakicidin A and B concentrations after 160 hours, determined by a two-tailed unpaired t-test.

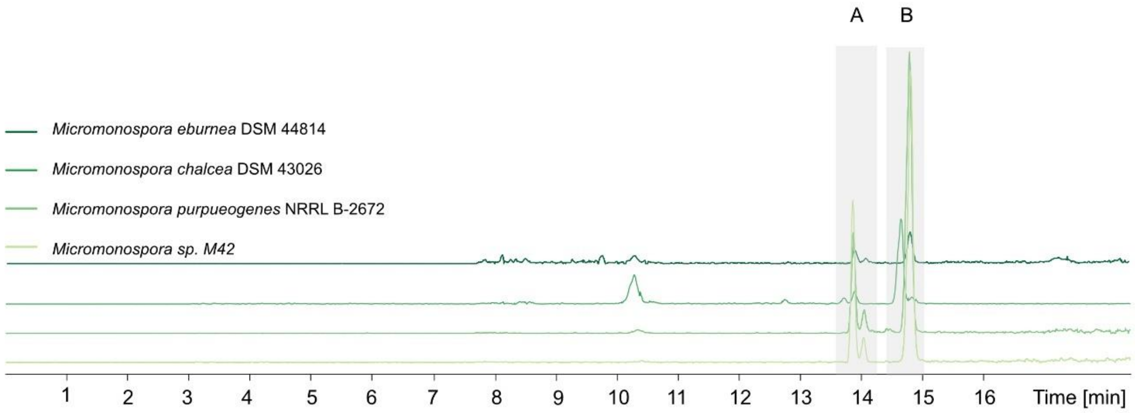

Fig. S10: Representative EIC of rakicidin A and B production at either 180 rpm or 20% pO_2. The EIC of 607.4 m/z (A) and 621.4 m/z (B) is indicated during the production of rakicidin A and B from the productions shown in **Fig. 5a** and **Fig. 6**. *Micromonospora* sp. M42 and *Micromonospora purpueogenes* NRRL B-2672 were cultivated in bioreactor with 20% pO_2, and *Micromonospora chalcea* DSM 43026 and *Micromonospora eburnea* DSM 44814 were cultivated in flasks at 180 rpm.

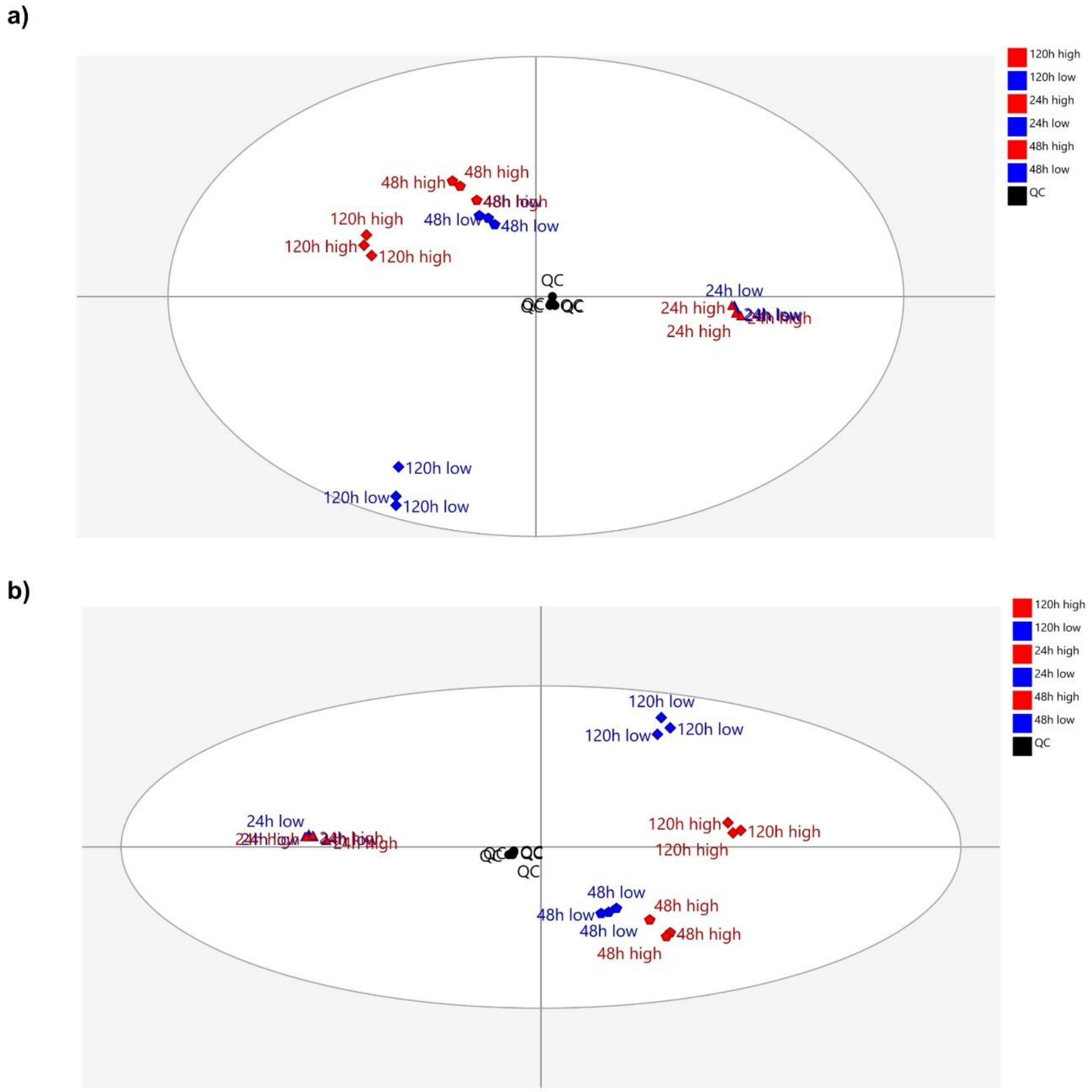

Fig. S11: PCA plot of data acquired in positive (a) and negative (b) electrospray ionization mode. Samples are extracted and analyzed after 24, 48, and 120 hours. Whether samples originate from low or high shaking speeds is indicated by the blue and red colors, respectively. There is an increasing difference between the two shaking speeds with increasing incubation times.

Table S1: Features detected and identified from *Micromonospora* sp. RV43. Features detected in positive or negative electrospray ionization (ESI) mode with their fold change (fc), p-value, and VIP score indicated after 48 and 120 hours, respectively. The features are sorted alphabetically into the subclasses of amino acid metabolism, nucleotides and derivatives, and others. fc indicates whether the metabolite is found in increased or decreased abundance at limited oxygen after 48 or 120 hours of cultivation (95 rpm, -O_2) compared to normal oxygen conditions (180 rpm, +O_2). The highlighted metabolites are statistically significantly abundant. Identification levels follow the Metabolomics Standards Initiative (MSI): Level 1 – confirmed by reference standard; Level 2 – putatively annotated (MS/MS and database).

Feature	ESI	m/z	RT (s)	identification	level	48 h			120 h		
						fc	p-value	VIP	fc	p-value	VIP
				Amino acid metabolism							
M90T36	positive	90.0543	36	Alanine	1	1.84	$8.16x10^{-2}$	3.71	0.63	$2.88x10^{-1}$	0.72

M175T34	positive	175.1180	34	Arginine	1	**0.69**	**1.65x10^{-3}**	**1.93**	1.44	1.10x10^{-2}	0.81
M176T37	positive	176.1016	37	Citrulline	1	**0.69**	**1.63x10^{-2}**	**1.32**	**0.40**	**5.37x10^{-3}**	**1.30**
M174T37	negative	174.0883	37	Cittruline	1	0.78	8.60x10^{-2}	0.92	**0.39**	**8.87x10^{-3}**	**1.39**
M230T44	positive	230.0951	44	L-Ergothioneine	1	**1.49**	**2.56x10^{-2}**	**3.06**	**3.02**	**2.53x10^{-4}**	**6.23**
M228T45	negative	228.0808	45	L-Ergothioneine	1	1.27	3.56x10^{-1}	0.28	**3.96**	**9.33x10^{-4}**	**1.35**
M148T36	positive	148.0596	36	Glutamate	1	0.69	1.41x10^{-1}	5.60	**0.63**	**2.95x10^{-3}**	**4.06**
M146T37	negative	146.0462	37	Glutamate	1	**0.67**	**3.10x10^{-3}**	**5.86**	**0.63**	**2.70x10^{-3}**	**5.20**
M147T35	positive	147.0753	35	Glutamine	1	**0.61**	**8.37x10^{-3}**	**1.27**	0.37	8.78x10^{-2}	1.23
M145T36	negative	145.0618	36	Glutamine	1	1.67	1.87x10^{-2}	0.80	0.43	7.28x10^{-3}	0.91
M132T114	positive	132.1016	114	iso-Leucine	1	**1.25**	**1.51x10^{-2}**	**6.09**	1.14	3.96x10^{-1}	0.75
M132T123	positive	132.1018	123	Leucine	1	1.00	1.00x10^{0}	5.32	1.38	6.52x10^{-2}	2.11
M147T29	positive	147.1118	29	Lysine	1	0.93	3.19x10^{-1}	1.02	**0.27**	**3.56x10^{-3}**	**3.97**
M150T66	positive	150.0579	66	Methionine	1	**1.43**	**1.45x10^{-3}**	**3.68**	1.27	5.43x10^{-2}	0.81
M217T58	positive	217.1287	58	N-Acetylarginine	1	0.94	7.93x10^{-2}	1.17	**0.52**	**3.33x10^{-2}**	**1.46**
M190T103	positive	190.0700	103	N-acetyl-glutamic acid	1	0.83	3.75x10^{-2}	1.05	0.72	6.46x10^{-2}	1.33
M188T106	negative	188.0570	106	N-acetyl-glutamic acid	1	1.04	1.08x10^{-1}	1.59	0.93	1.80x10^{-1}	1.44
M172T349	negative	172.0983	349	N-acetylleucine	1	1.09	1.53x10^{-1}	1.26	**6.38**	**6.74x10^{-4}**	**2.56**
M247T369	positive	247.1066	369	N-acetyltryptophane	1	1.12	2.38x10^{-1}	0.40	1.75	8.74x10^{-4}	0.92
M245T369	negative	245.0931	369	N-acetyltryptophane	1	**1.26**	**4.59x10^{-4}**	**1.63**	**1.72**	**4.35x10^{-3}**	**2.71**
M166T225	positive	166.0865	225	Phenylalanine	1	**1.25**	**8.83x10^{-3}**	**6.49**	**1.52**	**9.34x10^{-3}**	**2.27**
M116T41	positive	116.0704	41	Proline	1	1.04	1.93x10^{-2}	3.20	1.09	3.16x10^{-2}	1.03
M205T261	positive	205.0971	261	Tryptophane	1	**1.21**	**4.91x10^{-2}**	**3.90**	1.17	1.02x10^{-1}	0.83
M203T259	negative	203.0832	259	Tryptophane	1	**1.33**	**9.56x10^{-3}**	**4.88**	**1.51**	**4.19x10^{-4}**	**1.31**
M182T103	positive	182.0813	103	Tyrosine	1	1.16	5.98x10^{-4}	4.56	1.08	3.96x10^{-1}	0.85
M180T102	negative	180.0670	102	Tyrosine	1	**1.71**	**4.65x10^{-5}**	**3.01**	1.14	3.06x10^{-1}	0.31
M118T56	positive	118.0860	56	Valine	1	1.11	1.25x10^{-1}	3.67	0.97	4.84x10^{-1}	0.42
M162T39	positive	162.0750	39	2-aminoadipic acid	1	1.11	2.93x10^{-2}	0.68	**1.50**	**2.70x10^{-2}**	**1.59**
Nucleotides and derivatives											
M266T200	negative	266.0890	200	Adenosine	1	**1.72**	**6.44x10^{-3}**	**2.19**	1.86	1.68x10^{-1}	2.06
M348T66	positive	348.0711	66	AMP	1	**0.47**	**6.65x10^{-4}**	**8.40**	**0.51**	**1.55x10^{-3}**	**6.06**
M346T71	negative	346.0557	71	AMP	1	**0.46**	**3.23x10^{-3}**	**7.32**	**0.46**	**1.36x10^{-2}**	**5.46**
M324T47	positive	324.0580	47	CMP	1	**0.40**	**1.09x10^{-2}**	**1.57**	**0.26**	**3.91x10^{-2}**	**1.62**
M322T47	negative	322.0439	47	CMP	1	0.54	1.45x10^{-1}	0.97	0.36	7.04x10^{-2}	1.27
M244T55	positive	244.0917	55	Cytidine	1	**1.53**	**3.54x10^{-3}**	**1.18**	1.17	6.53x10^{-1}	0.45
M152T56	positive	152.0559	56	Guanine	1	1.18	1.95x10^{-1}	1.99	0.61	6.88x10^{-2}	1.84
M150T58	negative	150.0423	58	Guanine	1	1.42	6.94x10^{-2}	0.69	0.59	1.03x10^{-1}	0.47
M284T207	positive	284.0990	207	Guanosine	1	**1.88**	**3.72x10^{-3}**	**7.20**	1.11	4.65x10^{-1}	1.11
M282T209	negative	282.0845	209	Guanosine	1	**1.58**	**4.17x10^{-2}**	**5.45**	1.10	4.66x10^{-1}	0.98
M364T74	positive	364.0646	74	GMP	1	**0.33**	**7.09x10^{-4}**	**4.22**	**0.39**	**1.76x10^{-2}**	**2.00**
M362T80	negative	362.0500	80	GMP	1	**0.37**	**1.64x10^{-4}**	**6.59**	**0.32**	**2.26x10^{-2}**	**3.66**
M137T79	positive	137.0458	79	Hypoxanthine	1	**1.24**	**1.16x10^{-2}**	**5.54**	0.78	2.19x10^{-1}	2.04
M135T84	negative	135.0315	84	Hypoxanthine	1	1.51	7.12x10^{-3}	3.26	0.70	2.36x10^{-1}	0.99
M269T208	positive	269.0880	208	Inosine	1	**2.04**	**5.83x10^{-3}**	**6.38**	1.29	5.21x10^{-2}	1.98
M267T209	negative	267.0739	209	Inosine	1	**1.70**	**1.90x10^{-3}**	**9.40**	**1.41**	**1.67x10^{-2}**	**4.41**
M384T250	positive	384.1140	250	Succinyladenosine	1	1.14	7.08x10^{-2}	0.55	1.21	4.75x10^{-3}	0.60
M382T250	negative	382.0990	250	Succinyladenosine	1	1.19	2.73x10^{-2}	0.54	1.43	1.62x10^{-2}	0.73
M113T63	positive	113.0338	63	Uracil	1	0.95	3.40x10^{-1}	1.52	**0.51**	**3.49x10^{-3}**	**2.50**
M111T65	negative	111.0198	65	Uracil	1	1.07	1.16x10^{-1}	0.90	**0.50**	**6.59x10^{-3}**	**1.61**
M245T118	positive	245.0766	118	Uridine	1	**3.98**	**1.29x10^{-4}**	**6.20**	**2.95**	**1.77x10^{-2}**	**3.58**
M245T118	negative	243.0629	121	Uridine	1	**3.51**	**1.81x10^{-5}**	**13.45**	**2.30**	**5.59x10^{-3}**	**7.48**
M153T97	positive	153.0401	97	Xanthine	1	1.04	4.29x10^{-1}	1.05	1.16	2.98x10^{-2}	1.39
M151T101	negative	151.0265	101	Xanthine	1	1.18	1.56x10^{-2}	2.45	1.23	5.31x10^{-3}	1.86
M252T206	positive	252.1082	206	2-deoxyadenosine	1	**2.97**	**5.45x10^{-4}**	**1.80**	**2.34**	**4.87x10^{-2}**	**1.07**
M228T66	positive	228.0965	66	2-deoxycytidine	1	0.20	1.49x10^{-1}	2.07	5.64	3.08x10^{-1}	0.93
M385T150	positive	385.1290	150	5-adenosyl-homocysteine (SAH)	1	**0.67**	**1.93x10^{-4}**	**4.20**	**0.59**	**1.66x10^{-2}**	**3.07**
M383T153	negative	383.1138	153	5-adenosyl-homocysteine (SAH)	1	**0.72**	**4.39x10^{-5}**	**3.17**	**0.60**	**8.23x10^{-3}**	**2.66**
Others											
M564T698	positive	564.3640	698	BE-43547A	1	3.53	1.71x10^{-1}	1.59	**33.18**	**2.95x10^{-5}**	**10.21**
M562T698	negative	562.3477	698	BE-43547A	1	0.24	1.78x10^{-1}	1.17	**49.38**	**1.39x10^{-3}**	**7.03**
M578T719	positive	578.3792	719	BE-43547B	1	4.80	6.08x10^{-2}	5.10	**10.78**	**1.67x10^{-2}**	**11.18**
M576T719	negative	576.3631	719	BE-43547B	1	3.71	6.51x10^{-2}	2.44	**21.14**	**5.90x10^{-5}**	**7.50**
M592T734	positive	592.3946	734	BE-43547C	1	1.20	6.21x10^{-1}	0.55	**10.02**	**6.65x10^{-4}**	**9.40**
M590T734	negative	590.3782	734	BE-43547C	1	1.23	3.99x10^{-1}	0.23	**11.22**	**9.89x10^{-4}**	**2.63**
M258T36	positive	258.1101	36	Glycerophosphocholine	1	**1.20**	**6.95x10^{-3}**	**3.13**	**1.24**	**3.76x10^{-4}**	**2.30**
M487T78	positive	487.1604	78	Mycothiol	2	0.79	8.15x10^{-2}	3.75	1.41	6.47x10^{-2}	2.19
M485T83	negative	485.1446	83	Mycothiol	2	0.83	1.20x10^{-1}	6.23	1.25	1.84x10^{-1}	2.88
M664T110	positive	664.1177	110	NAD	1	**0.50**	**1.10x10^{-3}**	**9.61**	**0.52**	**1.36x10^{-3}**	**5.84**
M662T120	negative	662.1006	120	NAD	1	**0.41**	**7.29x10^{-5}**	**8.05**	**0.49**	**1.42x10^{-3}**	**4.78**
M666T215	positive	666.1316	215	NADH	1	**0.57**	**1.25x10^{-2}**	**3.60**	**0.42**	**6.52x10^{-3}**	**3.30**
M664T216	negative	664.1168	216	NADH	1	**0.50**	**4.66x10^{-2}**	**5.85**	**0.45**	**3.71x10^{-2}**	**4.46**
M124T69	positive	124.0388	69	Nicotinic acid	1	0.87	5.68x10^{-2}	1.62	**2.19**	**3.34x10^{-3}**	**2.75**
M290T95	positive	290.1342	95	Ophthalmic acid	1	0.97	3.41x10^{-1}	0.61	**1.37**	**4.54x10^{-4}**	**1.49**
M288T98	negative	288.1199	98	Ophthalmic acid	1	1.06	2.69x10^{-1}	0.74	1.21	3.29x10^{-3}	0.97
M130T59	positive	130.0862	59	Pipecolate	1	1.00	9.20x10^{-1}	1.51	**1.61**	**5.69x10^{-3}**	**6.22**
M377T324	positive	377.1447	324	Riboflavin	1	0.64	9.39x10^{-2}	1.41	**0.50**	**6.00x10^{-3}**	**1.69**
M375T324	negative	375.1298	324	Riboflavin	1	0.77	5.02x10^{-3}	0.81	0.74	1.67x10^{-1}	0.60

The milieu of the BE-43547 biosynthetic gene clusters has no known oxygen-dependent transcription factor binding sites.

Previous work by Urem *et al.* have shown that OsdR from *Streptomyces coelicolor* is a functional orthologue of the hypoxia-associated DevR from *Mycobacterium tuberculosis* and identified transcription factor binding site upstream of several stress related genes.[9] We analyzed the genomic area in the vicinity of several known APD-CLD biosynthetic gene clusters associated with BE-43547, rakicidin, and microtermolide. From **Fig. S12**, we see the core NRP/PK hybrid is conserved throughout all six producers as well as a rakicidin D BGC, and only partially conserved in the microtermolide BGC. Upon a more detailed inspection of each BGC using antiSMASH,[10] we employed the newly added feature for predicting transcription factor binding sites, including the one associated with OsdR. Looking at the BE-43547 producers (**a,b**), we were not able to identify any TFBS but found two genes that were identified as hypothetical proteins. Within these, no obvious conserved domains or similar conserved domains were identified, leaving us speculating about their function. In contrast, we found multiple predicted TFBS for the rakicidin producers (**c-g**), all being identified as cellobiose uptake repressors (medium confidence) except for the rakicidin D producer, where an antibiotic production activator was detected (AbrC3, strong confidence).

Only succeeding with an identification of medium confidence at the microtermolide BGC (**h**), we were not able to bioinformatically explain or identify any oxygen-dependent transcription factor binding sites in the BE-43547 BGC.

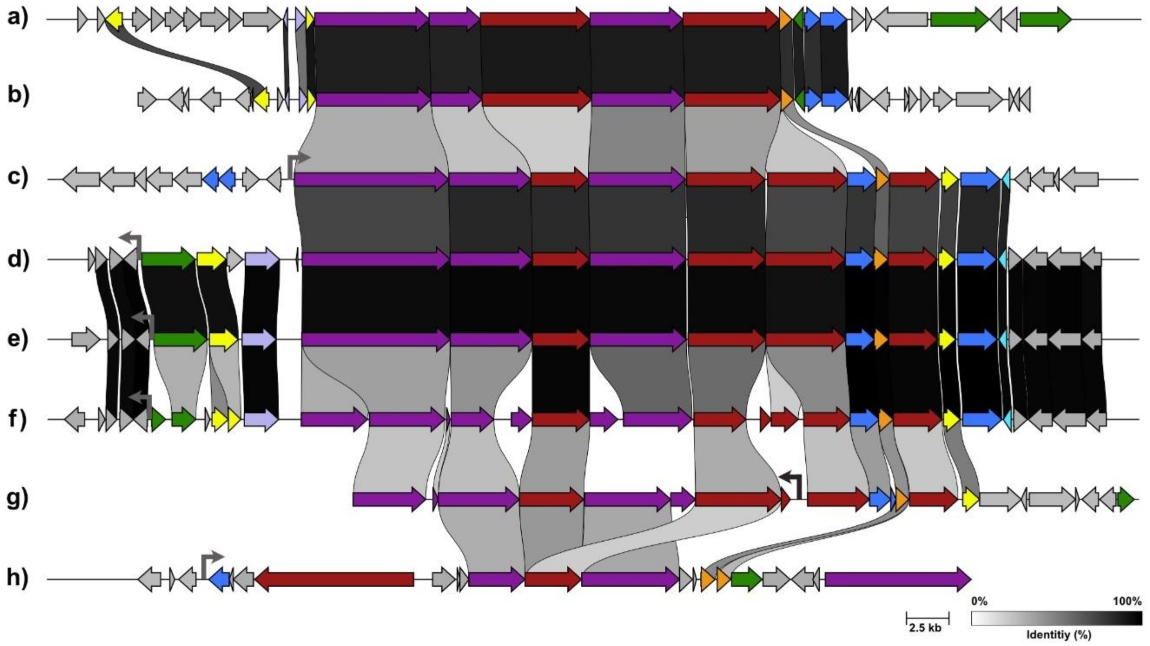

Fig. S12: BGCs associated with APD-CLD compounds and confirmed production. PKS, dark purple; NRPS, red, Thioesterases, orange; Transcriptional regulators, green; Transporters, blue; Oxidases, yellow; Epimerases, light blue; Unknown proteins, light purple. Predicted transcription factor binding sites (TFBSs) are indicated by vertical gray or black arrows on the BGCs, depending on whether their prediction confidence is medium or strong, respectively. The color of the areas in between the BGCs indicates the identity between the genes, going from white (0%) to black (100%). The BE-43547-associated BGC are shown for a) *Micromonospora* sp. RV43 and b) *Salinispora arenicola* CNR 107. The rakicidin-associated BGC are shown for c) *Micromonospora eburnea* DSM 44814, d) *Micromonospora chalcea* DSM 43026, e) *Micromonospora purpueogenes* NRRL B-2672, and f) *Micromonospora* sp. M42. The rakicidin D-associated BGC is shown for g) *Streptomyces lilacinus* NRRL B-1968. The microtermolide-associated BGC is shown for h) *Streptomyces* sp. MspMP-M5. The BGCs were analyzed with antiSMASH 7.0.1 (parameters: TFBS analysis, MIBiG, KnownClusterBlast) and compared by the web-based CAGECAT clinker (basic parameters).[11]

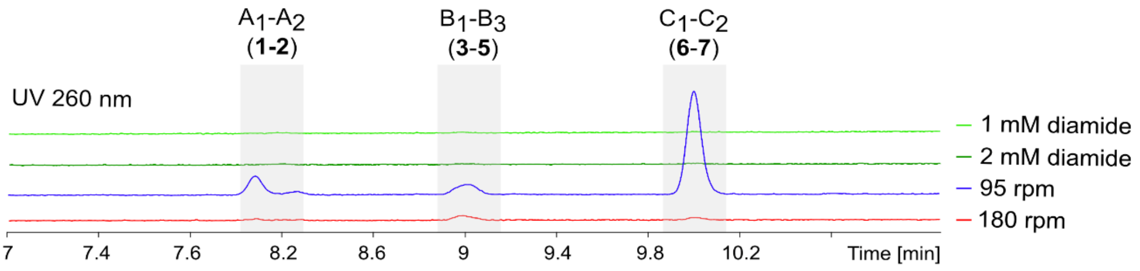

Fig. S13: Representative UV chromatograms at 260 nm after 7 days shaker flask cultivation of *Micromonospora* sp. RV43 with the addition 1,1-Azobis(N,N-dimethylformamide) (diamide, green) after 24 hours at 180 rpm shaking. Control flasks simulating low (95 rpm, blue) or high (180 rpm, red) oxygen conditions are shown. The retention times of the BE-43547 compounds are indicated in gray.

Table S2: Microorganisms used in this study and their source

**Strain**	**Source**
Acinetobacter baumannii DSM 30007	DSMZ
Enterobacter cloacae DSM 30054	DSMZ
Enterococcus faecium DSM 20477	DSMZ
Klebsiella oxytoca DSM 5175	DSMZ
Micromonospora chalcea DSM 43026	DSMZ
Micromonospora eburnea DSM 44814	DSMZ
Micromonospora purpueogenes NRRL B-2672	USDA-ARS Culture Collection (NRRL)
Micromonospora sp. M42	Kindly gifted by Prof. Russel T. Hill
Micromonospora sp. RV43	Kindly gifted by Prof. Dr. Ute Hentschel
Pseudomonas aeruginosa DSM 19880	DSMZ
Staphylococcus aureus DSM 20231	DSMZ
Streptomyces lilacinus NRRL B-1968	USDA-ARS Culture Collection (NRRL)

Table S3: Media recipes used in this study

**Medium**			
LB	Luria Bertani broth (1 L)	25 g<br>15 g	Premixed LB<br>Agar if needed
TSB	Tryptic soy broth (1 L)	30 g	Premixed TSB
MHB	Mueller-Hinton broth (1 L)	21 g	Premixed MHB
Minimal M9	Minimal M9 (1 L)	11.28<br>Add dH_2O<br>Autoclaved	M9 salts<br>pH adjusted to 7.4
		1 mL<br>1 mL<br>6.74 mL<br>306 µL<br>1 mL	$CaCl_2$<br>$MgSO_4$<br>Thiamine-HCl<br>Nicotinamide<br>Trace element solution
	If growth-promoting	10 g<br><br>20 mL	Casamino acids before autoclave<br>Glucose solution
	$CaCl_2$ (10 mL)<br>$MgSO_4$ (10 mL)<br>Thiamine-HCl (50 mL)<br>Nicotinamide (50 mL)<br>Glucose solution (50 mL)	147 mg<br>4.9 g<br>2.5 g<br>1 g<br>25 g	$CaCl_2$<br>$MgSO_4$<br>Thiamine-HCl<br>Nicotinamide<br>Glucose
	Trace element solution (1 L)	2 g<br>250 mg<br>100 mg<br>70 mg<br>6 mg<br>40 mg<br>70 mg<br>2 mg	$FeCl_2 * H_2O$<br>$CoCl_2 * 6 H_2O$<br>$MnCl_2 * 4 H_2O$<br>$ZnCl_2$<br>H_3BO_3<br>$Na_2MoO_4 *2H_2O$<br>$NiCl_2 * 6H_2O$<br>$CuCl_2 *2 H_2O$

		60 mg<br>6 mg<br>10 mL	$AlCl_3$ *6 H_2O<br>$NaWo_4$* 2*H_2O<br>HCl (37%)
ISP-2 with marine salts	ISP-2 with marine salts (1 L)	10 g<br><br>4 g<br>4 g<br>22 g<br>Add dH_2O<br>Autoclaved	Malt extract<br><br>Glucose<br>Yeast extract<br>Marine salts<br>pH adjusted to 7.2
M8 with marine salts	M8 with marine salts (1 L)	20 g<br>10 g<br>2 g<br>2 g<br>4 g<br>3 g<br>22 g<br>Add dH_2O<br>Autoclaved	Soluble starch<br>Glucose<br>Yeast extract<br>Meat extract<br>Hydrolyzed casein<br>$CaCO_3$<br>Instant Ocean (marine salt)<br>pH adjusted to 7.0
A1M1	A1M1 (1 L)	5 g	Soluble starch<br>Add 50 mL dH_2O to get it into solution. Then add 950 mL of boiling water to fully dissolve before adding the remaining components
		2 g<br>2 g<br>22 g<br>Add dH_2O<br>Autoclaved	Peptone from casein<br>Yeast extract<br>Marine salts<br>pH adjusted to 7.2
Bioreactors	Feed 1 (1 L)	14 g<br>4 g<br>22 g<br>Add dH_2O<br>Autoclaved	Glucose<br>Yeast extract<br>Marine salts
	Feed 2 (1 L)	10 g	Soluble starch<br>Prepared as described above
		10 g<br>4 g<br>4 g<br>2 g<br>22 g	Malt extract<br>Glucose<br>Yeast extract<br>Peptone from soy<br>Marine salts

Table S4: BE-43547 concentrations [mg/L] from each biological replicate from Fig. 3c. At each time-point, extractions were carried out in technical duplicates, and the data from these are shown below. The color indicates whether the bioreactor

replicate had a setting of 5% (blue) or 20% (red) pO_2 after 48-72 hours. The rep. 7 bioreactor did not keep its pO_2-setting at 20%, and is therefore not considered in either of these categories.

Batch time (h)	Rep. 1		Rep. 2		Rep. 3		Rep. 4		Rep. 5		Rep. 6		Rep. 7 (black filled circles)	
**24**	0.0	0.0	0.0	0.0	0.0	0.0	0.0	0.0	0.0	0.0	0.0	0.0	0.0	0.0
**48**	0.0	0.0	0.0	0.0	0.0	0.0	0.0	0.0	0.0	0.0	0.0	0.0	0.0	0.0
**72**	0.0	0.0	0.0	0.0	0.0	0.0	0.0	0.0	0.0	0.0	0.0	0.0	0.0	0.0
**96**	0.0	0.0	5.1	6.5	0.0	0.0	0.0	0.0	0.0	0.0	0.0	0.0	0.0	0.0
**120**	11.7	11.9	5.3	11.7	7.4	7.4	0.0	0.0	0.0	0.0	3.4	3.2	0.0	0.0
**144**	18.6	17.6	23.9	23.3	24.7	24.4	0.0	0.0	0.0	0.0	0.0	3.3	0.0	0.0
**168**	26.2	26.0	26.1	26.7	32.2	33.6	0.0	0.0	3.0	3.3	2.8	3.0	8.8	9.4
**192**	33.0	32.0	30.0	27.3	38.2	40.1	0.0	0.0	3.2	3.0	2.9	2.9	9.4	9.7
**216**	30.2	29.0	-	-	-	-	-	-	2.8	3.0	0.0	2.8	11.4	11.6

Table S5: BE-43547 concentrations [mg/L] from each biological replicate from Fig. 4b. At each time-point, extractions were carried out in technical duplicates, and the data from these are shown below. The color indicates whether the shaker flask replicate had a setting of 95 rpm (blue) or 180 rpm (red) after 24 hours.

Batch time (h)	Rep. 1		Rep. 2		Rep. 3		Rep. 4		Rep. 5		Rep. 6	
**24**	0.0	0.0	0.0	0.0	0.0	0.0	0.0	0.0	0.0	0.0	0.0	0.0
**48**	0.0	0.0	0.0	0.0	0.0	0.0	0.0	0.0	0.0	0.0	0.0	0.0
**72**	0.0	0.0	0.0	0.0	0.0	0.0	0.0	0.0	0.0	0.0	0.0	0.0
**96**	4.7	1.7	12.7	7.2	7.1	8.8	0.0	0.0	0.0	0.0	0.0	0.0
**120**	2.8	4.8	11.9	12.8	13.6	11.9	0.0	0.0	0.0	0.0	0.0	0.0
**144**	7.3	9.3	15.1	14.2	12.6	15.0	0.0	0.0	0.0	0.0	0.0	0.0
**192**	7.0	6.5	10.1	9.9	9.1	10.1	0.0	0.0	0.0	0.0	0.0	0.0

Table S6: Rakicidin A and B concentrations [mg/L] from each biological replicate from Fig. 5a. At each time-point, extractions were carried out in technical duplicates, and the data from these are shown below. The color indicates whether the bioreactor replicate had a setting of 5% (blue) or 20% (red) pO_2 after 60 hours.

Batch time (h)	Rep. 1		Rep. 2		Rep. 3		Rep. 4	
**24**	0.0	0.0	0.0	0.0	0.0	0.0	0.0	0.0
**48**	0.0	0.0	0.0	0.0	0.0	0.0	0.0	0.0
**72**	0.0	0.0	5.5	6.0	15.4	15.4	11.6	10.9
**96**	5.5	5.4	6.4	5.0	18.5	18.2	3.08	12.9
**120**	6.1	5.7	7.1	7.3	19.2	19.1	14.1	13.9
**144**	6.4	6.1	7.0	6.5	18.0	18.6	14.0	11.5

Table S7: BE-43547 concentrations [mg/L] from each biological replicate from Fig. S7. At each time-point, extractions were carried out in technical duplicates, and the data from these are shown below. The color indicates whether the bioreactor replicate had a setting of 5% (blue) or 20% (red) pO_2 after 60 hours.

Batch time (h)	Rep. 1		Rep. 2		Rep. 3		Rep. 4	
**24**	0.0	0.0	0.0	0.0	0.0	0.0	0.0	0.0
**48**	0.0	0.0	0.0	0.0	0.0	0.0	0.0	0.0
**60**	0.0	0.0	0.0	0.0	0.0	0.0	0.0	0.0
**85**	6.1	2.4	0.0	0.0	0.0	2.5	0.0	0.0
**109**	11.9	13.3	5.5	4.7	1.3	0.0	0.0	0.0
**136**	25.4	28.0	13.5	14.6	4.6	2.3	4.4	2.8
**158**	38.6	40.3	27.4	26.0	0.0	3.7	8.1	6.6
**182**	35.5	28.7	25.7	31.6	2.9	6.5	6.6	13.4
**206**	36.5	34.8	26.6	25.0	4.5	6.6	3.3	5.0

Table S8: Rakicidin A and B concentrations [mg/L] from each biological replicate from Fig. S9. At each time-point, extractions were carried out in technical duplicates, and the data from these are shown below. The color indicates whether the bioreactor replicate had a setting of 5% (blue) or 20% (red) pO_2 after 60 hours.

Batch time (h)	Rep. 1		Rep. 2		Rep. 3		Rep. 4	
24	0.0	0.0	0.0	0.0	0.0	0.0	0.0	0.0
36	0.0	0.0	-	-	0.0	0.0	-	-
48	-	-	0.0	0.0	-	-	0.0	0.0
60	5.9	5.5	-	-	7.6	7.1	-	-
72	-	-	0.0	0.0	-	-	0.0	0.
90	0.0	0.0	-	-	7.6	7.8	-	-
96	-	-	0.0	0.0	-	-	2.1	2.2
110	0.0	0.0	-	-	8.2	7.9	-	-
120	-	-	0.0	0.0	-	-	7.1	7.8
134	0.0	0.0	-	-	12.1	11.5	-	-
160	0.0	0.0	0.0	0.0	14.4	12.8	4.4	6.0

References

[1] N. L. Villadsen, K. M. Jacobsen, U. B. Keiding, E. T. Weibel, B. Christiansen, T. Vosegaard, M. Bjerring, F. Jensen, M. Johannsen, T. Tørring, T. B. Poulsen, "Synthesis of ent-BE-43547A1 reveals a potent hypoxia-selective anticancer agent and uncovers the biosynthetic origin of the APD-CLD natural products" *Nature Chem* **2017**, *9*, 264–272.

[2] K. D. McBrien, R. L. Berry, S. E. Lowe, K. M. Neddermann, I. Bursuker, S. Huang, S. E. Klohr, J. E. Leet, "Rakicidins, new cytotoxic lipopeptides from Micromonospora sp. fermentation, isolation and characterization" *J Antibiot (Tokyo)* **1995**, *48*, 1446–1452.

[3] W. Landwehr, S. Karwehl, P. J. Schupp, P. Schumann, J. Wink, "Biological Active Rakicidins A, B and E produced by the Marine Micromonospora sp. Isolate Guam1582" *AIBM* **2016**, *1*, DOI 10.19080/AIBM.2016.01.555558.

[4] L. Xie, L. Chen, Y. Wei, N. Chen, T. Wu, J. Zhou, H. Jiang, F. Lin, "Design, Synthesis and Biological Evaluation of Novel PEG-Rakicidin B1 Hybrid as Clostridium difficile (CD) Targeted Anti-Bacterial Agent" *Molecules* **2023**, *28*, 6152.

[5] M. Igarashi, T. Shida, Y. Sasaki, N. Kinoshita, H. Naganawa, M. Hamada, T. Takeuchi, "Vinylamycin, a new depsipeptide antibiotic, from Streptomyces sp." *J Antibiot (Tokyo)* **1999**, *52*, 873–879.

[6] L. Chen, W. Zhao, H.-L. Jiang, J. Zhou, X.-M. Chen, Y.-Y. Lian, H. Jiang, F. Lin, "Rakicidins G - I, cyclic depsipeptides from marine *Micromonospora chalcea* FIM 02-523" *Tetrahedron* **2018**, *74*, 4151–4154.

[7] S. Kitani, T. Ueguchi, Y. Igarashi, K. Leetanasaksakul, A. Thamchaipenet, T. Nihira, "Rakicidin F, a new antibacterial cyclic depsipeptide from a marine sponge-derived Streptomyces sp." *J Antibiot* **2018**, *71*, 139–141.

[8] J. P. Torres, Z. Lin, D. S. Fenton, L. U. Leavitt, C. Niu, P.-Y. Lam, J. M. Robes, R. T. Peterson, G. P. Concepcion, M. G. Haygood, B. M. Olivera, E. W. Schmidt,

"Boholamide A, an APD-Class, Hypoxia-Selective Cyclodepsipeptide" *J. Nat. Prod.* **2020**, *83*, 1249–1257.
[9] M. Urem, T. van Rossum, G. Bucca, G. F. Moolenaar, E. Laing, M. A. Świątek-Połatyńska, J. Willemse, E. Tenconi, S. Rigali, N. Goosen, C. P. Smith, G. P. van Wezel, "OsdR of Streptomyces coelicolor and the Dormancy Regulator DevR of Mycobacterium tuberculosis Control Overlapping Regulons" *mSystems* **2016**, *1*, 10.1128/msystems.00014-16.
[10] K. Blin, S. Shaw, H. E. Augustijn, Z. L. Reitz, F. Biermann, M. Alanjary, A. Fetter, B. R. Terlouw, W. W. Metcalf, E. J. N. Helfrich, G. P. van Wezel, M. H. Medema, T. Weber, "antiSMASH 7.0: new and improved predictions for detection, regulation, chemical structures and visualisation" *Nucleic Acids Res.* **2023**, *51*, W46–W50.
[11] M. van den Belt, C. Gilchrist, T. J. Booth, Y.-H. Chooi, M. H. Medema, M. Alanjary, "CAGECAT: The CompArative GEne Cluster Analysis Toolbox for rapid search and visualisation of homologous gene clusters" *BMC Bioinformatics* **2023**, *24*, 181.
